# Microbiota and Transcriptomic Effects of an Essential Oil Blend and Its Delivery Route Compared to an Antibiotic Growth Promoter in Broiler Chickens

**DOI:** 10.3390/microorganisms10050861

**Published:** 2022-04-21

**Authors:** Samson Oladokun, K. Fraser Clark, Deborah I. Adewole

**Affiliations:** Department of Animal Science and Aquaculture, Faculty of Agriculture, Dalhousie University, Truro, NS B2N 5E3, Canada; samson.oladokun@dal.ca (S.O.); fraser.clark@dal.ca (K.F.C.)

**Keywords:** microbiota, transcriptomics, essential oil, delivery routes, antibiotics, broiler chickens

## Abstract

This study evaluated the effect of the delivery of a commercial essential oil blend containing the phytonutrients star anise, cinnamon, rosemary, and thyme oil (via different routes) on broiler chickens’ ileal and ceca microbiota and liver transcriptome compared to an antibiotic growth promoter. Eggs were incubated and allocated into three groups: non-injected, in ovo saline, and in ovo essential oil. On day 18 of incubation, 0.2 mL of essential oil in saline (dilution ratio of 2:1) or saline alone was injected into the amnion. At hatch, chicks were assigned to post-hatch treatment combinations: (A) a negative control (corn-wheat-soybean diet), (B) in-feed antibiotics, (C) in-water essential oil (250 mL/1000 L of drinking water), (D) in ovo saline, (E) in ovo essential oil, and (F) in ovo essential oil plus in-water essential oil in eight replicate cages (six birds/cage) and raised for 28 days. On days 21 and 28, one and two birds per cage were slaughtered, respectively, to collect gut content and liver tissues for further analysis. Alpha and beta diversity differed significantly between ileal and ceca samples but not between treatment groups. In-feed antibiotic treatment significantly increased the proportion of specific bacteria in the family *Lachnospiraceae* while reducing the proportion of bacteria in the genus *Christensenellaceae* in the ceca, compared to other treatments. Sex-controlled differential expression of genes related to cell signaling and tight junctions were recorded. This study provides data that could guide the use of these feed additives and a foundation for further research.

## 1. Introduction

Over the years, the sub-therapeutic supplementation of antibiotic growth promoters (AGPs) has been used to preserve gut health, intestinal microbiota balance, and growth performance in the poultry industry [1]. This trend has now triggered both consumer and public health concerns bordering on the emergence of antibiotic resistance and antibiotic residues in the food chain [2,3]. Accordingly, a few country-specific restrictions on the use of AGPs are already in place, including in the EU [4], the US [5], and Canada [6]. The potential elimination of AGPs could exacerbate the risks of intestinal dysbiosis and bacterial diseases in poultry [7]. In the post-AGP era, understanding the complex interplay between the host and its intestinal microbiome signifies a critical step to achieving optimum gut health and intestinal microbiota balance in poultry.

The gastrointestinal tract (GIT) of poultry, populated by microorganisms in constant interaction with the host and digesta, is known to play a critical role in the host’s growth and health. It is now evident that the proliferation of a balanced and beneficial gut microbiota population is vital to ensuring host protection against pathogenic bacteria and enhancing gut integrity and immunity [8,9,10]. Several factors, including environmental stressors [11,12,13], bird age [14], and nutrition [15,16,17] can modify the gut microbiota profile. Of all these factors, nutrition (including the type of diet and time of feeding) has been regarded as the main factor influencing poultry gut microbiota dynamics [9]. Given this modulatory role, several feed additives, including probiotics, prebiotics, organic acids, exogenous enzymes, and essential oils, are being investigated as potential alternatives to AGPs in the poultry industry [9].

Essential oils (EOs) are mostly plant extracts with mixtures of phytochemical compounds like thymol, carvacrol, and eugenol [18]. To explore a synergistic effect, commercial combinations and blends of several EO types are becoming increasingly popular. Several in vitro studies have highlighted the antibacterial, antiviral, antifungal, antimycotic, antiparasitic, antioxidant, anti-inflammatory, anti-toxigenic, and immune-regulating properties of EOs [19,20,21]. However, in vivo results on the effect of EO on chicken microbiota are somewhat inconsistent. While EO blends have been reported to reduce the relative abundance of pathogenic bacteria like *Escherichia coli* [22,23], Salmonella [24], and *Clostridium perfringens* [25] in broiler chickens, a few studies have equally reported no effect of EO supplementation on gut commensal bacteria [24,26,27]. These inconsistencies in the efficacy of EOs could be associated with the limitations that characterize their mode of delivery [28,29], as most EOs are conventionally supplied via feed or water to poultry birds. These conventional routes expose EOs to potential thermal instability, especially during feed milling processes like pelleting [30] and negative interaction with other feed additives like oligosaccharides and coccidiostats [31,32]. The success of in-water EO supplementation will depend on the water quality and the quality of the chick watering device. In-water EO delivery also has the potential to promote wet feather risks and other welfare issues.

The delivery of EO via the in ovo route presents a viable means to overcome the identified challenges that characterize conventional delivery routes (i.e., in feed and in water). In ovo delivery of bioactive substances has been defined as “the direct inoculation of bioactive substances to the developing embryo to elicit superior lifelong effects, while considering the dynamic physiology of the chicken embryo” [33]. The in ovo delivery route offers an economic advantage, as low doses of bioactive substances are required to initiate long-term performance effects in the birds [33,34]. It offers the opportunity to stimulate the colonization of the embryonic gut with beneficial microbiota very early on, rather than trying to alter an already established microbiota community in later life [35]. Additionally, it is yet to be known if an additive benefit exists from the successive delivery of EOs via the in ovo and continuous in-water delivery routes. This study is thus interested in evaluating if such an effect exists in the broiler chicken microbiota and liver transcriptome.

Studies have also suggested that microbial community might vary depending on the segment of the small intestine considered [36,37,38]. In addition to the reported microbiota modifying effect, EOs can also influence the expression of several genes involved in de novo fat synthesis and deposition [39] as well as antioxidant activity [40]. Studies involving the liver transcriptome of EO-fed birds have also reported the enrichment of Gene Ontology Consortium (GO) terms associated with performance and metabolism [39], as well as a higher expression of antioxidant genes [41]. The liver remains a good candidate tissue to study the transcriptomic effect of EO supplementation, as it is involved in several metabolic functions, including carbohydrate, protein, and lipid metabolism; bile secretion; and immune defense, among others [42]. Additionally, the combination of modern molecular biological techniques, such as 16S ribosomal RNA (16S rRNA) gene sequencing and RNA sequencing (RNA-seq) technology, could help unravel the precise mechanism underpinning the delivery of EOs. Most studies on EO delivery have mainly focused on low throughput gene expression analysis and bird performance evaluation [43,44]. Accordingly, the objective of this study was to evaluate the effect of a commercial EO blend containing star anise, cinnamon, rosemary, and thyme oil delivered via in-water and in ovo routes on broiler chickens’ ileal and ceca microbiota, ceca short-chain fatty acid concentration, and liver transcriptome as compared to an in-feed antibiotic growth promoter.

## 2. Materials and Methods

### 2.1. Ethics Statement

The experiment was carried out at the hatchery facility of the Agricultural Campus of Dalhousie University and the broiler rearing facility of the Atlantic Poultry Research Center, Dalhousie Faculty of Agriculture. All experimental procedures were approved by the Animal Care and Use Committee of Dalhousie University (Protocol number: 2020-035), in accordance with the guidelines of the Canadian Council on Animal Care [45].

### 2.2. Egg Incubation and In Ovo Injection Procedure

A total of 670 hatching eggs with an average weight 77.87 ± 2.43 g (mean ± SE) from 41-week-old Cobb 500 broiler breeders were sourced from Synergy hatchery, Nova Scotia, Canada. Eggs were incubated in a ChickMaster single-stage incubator (ChickMaster G09, Cresskill, NJ, USA) under standard conditions (37.5 °C and 55% relative humidity) from embryonic days (EDs) 1 to 17, and then to an average of 32 °C and 68% from EDs 18 to 21. Incubators were preheated for 24 h prior to setting eggs to ensure that proper temperature and humidity were stable. Egg trays were turned on a 90° arc four times an hour from the time of setting until ED 18. Eggs were arranged in 6 replicate trays inside the incubator, with each tray containing 96 eggs. On ED 12, eggs were candled, and in-fertile eggs were disposed of, leaving a total of 576 eggs for the trial. The remaining eggs were subsequently assigned to one of three treatment groups: (1) non-injected eggs (control, 288 eggs), (2) in ovo saline group (96 eggs, injected with 0.2 mL of physiological saline, i.e., 0.9% NaCl), (3) in ovo essential oil group (192 eggs, injected with 0.2 mL of a saline and essential oil blend mixture at a dilution ratio of 2:1). The essential oil utilized in this study is a commercial blend (Probiotech International Inc., St Hyacinthe, QC, Canada) containing the phytonutrients star anise, cinnamon, rosemary, and thyme oil. The EO blend is registered by Health Canada as a veterinary health product (VHP). On ED 18, eggs were injected according to the procedure described by Oladokun et al. [46] with slight modifications. Briefly, this involved disinfecting the eggs with 70% ethanol-dipped swabs and using an 18-gauge needle to carefully punch the shell at the center of the air cell (the blunt end). The injected EO was then delivered to the amnion using a self-refilling injector (Socorex ultra-1810.2.05005, Ecublens, Switzerland) equipped with a 22-gauge needle (injection needle length—3 cm) at a 45-degree angle. After in ovo injection, the injection sites were sealed with sterile paraffin and eggs were placed back in the incubator. The non-injected eggs were also taken out and returned to the incubator simultaneously as other injected treatment groups.

### 2.3. Birds, Housing, and Diets

**Figure 1:**
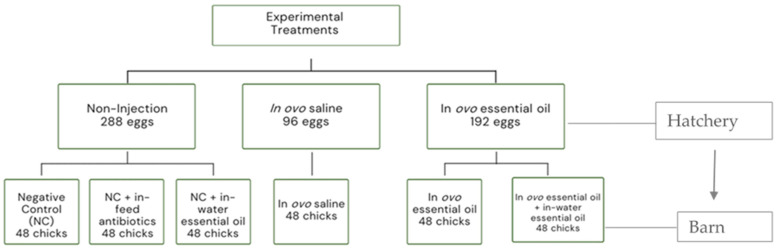
Schematic presentation of experimental structure in the hatchery and barn.

Hatchlings were weighed and randomly assigned to 6 new treatment groups (Figure 1). Chicks (straight run) from the initial non-injection group were randomly allocated into 3 new treatment groups consisting of (A) chicks fed a basal corn-soybean meal-wheat–based diet (negative control treatment, NC), (B) chicks fed NC + 0.05% bacitracin methylene disalicylate (in-feed antibiotics), and (C) chicks supplied the same commercial blend of EOs as earlier described via the water route (in-water essential oil) at the recommended dosage of 250 mL/1000 L of drinking water. The initial in ovo saline and in ovo essential oil groups were placed on the control diet to form treatments (D) (in ovo saline treatment) and (E) (in ovo essential oil treatment), respectively. The last treatment group, (F), consisted of chicks from the in ovo essential oil treatment group also supplied EO via the water route (in ovo + in-water essential oil treatment). All treatment groups had 48 birds each. Birds were placed in battery cages (0.93 m × 2.14 m), there were 6 birds per cage, and 8 replicate cages per treatment. Birds were reared for 28 d under uniform controlled environmental conditions in line with Cobb Broiler Management Guide recommendations. The room temperature was set at 31 °C on day 0 and gradually reduced to 23 °C on day 28, and relative humidity ranged between 45 and 55%. The ingredient and nutritional compositions of the basal diet used in the study are available in Oladokun et al. [47] and Supplementary Table S1. Birds were provided with feed and water ad libitum and diets were fed as mash throughout the rearing period which included the starter (0–14 d) and grower (15–28 d) phases. Diets met or exceeded the NRC [48] nutritional requirements for broiler chickens.

### 2.4. Sample Collection

On day 21, 1 bird per cage (8 replicate birds per treatment group) was randomly selected, weighed, and euthanized by electrical stunning and exsanguination. After slaughter, the small intestinal segment—the ileum (1.5-cm length mid-way between Meckel’s diverticulum and the ileocecal junction)—was longitudinally opened, and digesta content was collected into microcentrifuge tubes. Aside from being the most studied small intestinal segments, the ileum microbiota was evaluated because reported trends suggest increasing microbial density in the distal region of the small intestine compared to the proximal regions as a result of longer digesta transit times [17,37].

Similarly, on day 28, 2 birds per cage (16 replicate birds per treatment group) were randomly selected and euthanized by electrical stunning and exsanguination. After euthanasia of the birds, digesta content from the pair of ceca was mixed and divided into two subsamples. One part was stored in plastic RNase- and DNase-free tubes placed in liquid nitrogen to analyze gut microbiota. The other part was placed in bio-freeze kits (Alimetric Diagnostics, Espoo, Finland) for the determination of short-chain fatty acids following published protocols [46]. Liver tissues (50–100 mg) using 1 mL TRIzol™ (Qiagen, Hilden, Germany) from 8 replicate birds/treatment were also rapidly collected on day 28 and promptly frozen in liquid nitrogen. All samples were stored at −80 °C until further analysis.

### 2.5. DNA Extraction, Qualification, Library Preparation, and Sequencing

The Qiagen DNeasy^®^ PowerSoil Pro Kit (50) (Cat. No./ID: 47014) was used to extract DNA from both ileal and ceca digesta contents. Digesta contents were allowed to thaw briefly at room temperature before subsequent DNA extraction, following the manufacturer’s protocol. Briefly, 250 mg of digesta content was added to PowerBead Pro Tubes and then subjected to cell lysing steps involving vortexing and centrifugation. The retrieved lysate was then captured onto an MB Spin Column, followed by a series of purification and centrifugation steps. The MB Spin Column was then carefully placed into the provided 1.5 mL elution tubes from which extracted DNA was recovered. The concentration and purity of extracted DNA were subsequently determined by spectrophotometry (Nanodrop ND1000, Thermo Scientific, Waltham, MA, USA). Extracted DNA samples (volume—50 µL, concentration—10–200 ng/µL) were then sent to the Integrated Microbiome Resource (IMR), located at Dalhousie University in Halifax, Nova Scotia, for library preparation and sequencing. Libraries of the V4–V5 hypervariable region of the 16S rRNA gene were prepared using universal primers 515 F (Illumina adapters + 5′GTGYCAGCMGCCGCGGTAA3′) and 926 R (Illumina adapters + 5′CCGYCAATTYMTTTRAGTTT3′) following protocols described by Comeau et al. [49]. Each sample was amplified with a different combination of index barcodes to allow for sample identification after multiplex sequencing. Library preparation and sequencing for all samples were performed with the Illumina MiSeq at the Integrated Microbiome Resource (http://imr.bio/, accessed on 15 July 2021) of Dalhousie University.

### 2.6. Short-Chain Fatty Acid Concentration and Total Bacteria Density

Ceca samples were collected using BioFreeze™ sampling kits (Alimetrics Diagnostics Ltd., Espoo, Finland) following the manufacturer’s protocol. Samples were then subsequently submitted to Alimetrics Diagnostics 20007-1 (Espoo, Finland) for both SCFA concentration and total bacterial density quantification. The SCFA profiles were analyzed by gas chromatography (Agilent Technologies, Santa Clara, CA, USA) using pivalic acid as an internal standard. The acids quantified included acetic, propionic, butyric, valeric, and lactic acids. To quantify the total bacteria density, submitted samples were initially washed to remove solid particles and complex polysaccharides that may disturb subsequent DNA purification processes and downstream qPCR applications. The liquid phase was subjected to differential centrifugation for collecting the bacterial cells. The cell walls of the microbial cells were disrupted, and the chromosomal DNA was quantitatively extracted and purified using optimized protocol (Alimetrics Diagnostics 20007-1, Espoo, Finland). All measurements were performed with 16 replicates per treatment group.

### 2.7. RNA Extraction, Qualification, Library Preparation, and Sequencing

Total RNA in liver tissues was extracted on a QIAcube Connect using RNeasy Plus Universal Mini Kit (Qiagen, Cat. No. ID: 73404) following the manufacturer’s instructions after disruption and homogenization were performed with a TissueLyser system. RNA elution volume was 30 µL. Total RNA was quantified, and its integrity was assessed on a LabChip GXII (PerkinElmer). Libraries were generated from 250 ng of total RNA and mRNA enrichment was performed using the NEBNext Poly(A) Magnetic Isolation Module (New England BioLabs). cDNA synthesis was achieved with the NEBNext RNA First-Strand Synthesis and NEBNext Ultra Directional RNA Second Strand Synthesis Modules (New England BioLabs). The remaining library preparation steps were performed using the NEBNext Ultra II DNA Library Prep Kit for Illumina (New England BioLabs). Adapters and PCR primers were purchased from New England BioLabs. Libraries were quantified using the Kapa Illumina GA with Revised Primers-SYBR Fast Universal kit (Kapa Biosystems). Average fragment size was determined using a LabChip GXII (PerkinElmer) instrument. The libraries were normalized, pooled, and then denatured in 0.05 N NaOH and neutralized using HT1 buffer. The pool was loaded at 225 pM on an Illumina NovaSeq S4 lane using Xp protocol as per the manufacturer’s recommendations. The run was performed for 2 × 100 cycles (paired-end mode). A phiX library was used as a control and mixed with libraries at 1% level. Base calling was performed with RTA v3.4.4. The program bcl2fastq2 v2.20 was then used to demultiplex samples and generate fastq reads.

### 2.8. Bioinformatics and Statistical Analysis

The analysis of microbiota data was carried out using the Microbiome Helper pipeline (https://github.com/LangilleLab/microbiome_helper/wiki, accessed on 29 July 2021), based on QIIME2. This uses amplicon sequence variants (ASVs) created with Deblur. Primer sequences were removed from sequencing reads using cutadapt (v 1.14) [50], and primer-trimmed files were imported into QIIME2 (v. 2019.4.0) [51]. Reads (forward and reverse paired ends) were joined using VSEARCH (v 2.9.0) [52] and inputted into Deblur [53] to correct reads and obtain amplicon sequence variants (ASVs). Taxonomic assignment was performed with the SILVA database (v.1.3.2) using a naive Bayes approach implemented in the scikit learn Python library [49]. Rarefaction curves were used to examine the individual alpha diversity for all samples (with the default observed OTUs as the metric). Alpha diversity comparisons for the treatments were explored using boxplots and the Kruskal–Wallis statistical test set at *p* < 0.05. Beta diversity was visualized using weighted UniFrac PCoA plots. The relative abundance at different taxonomic levels was visualized using stacked bar charts, while significant microbiota proportions were determined in the Statistical Analysis of Metagenomic Profiles (STAMP) software [54] with an ANOVA test using the Benjamin–Hochberg false discovery rate as multiple test correction and then sorting by Corrected *p*-value (*p* < 0.05). Data on SCFA concentrations and total bacteria density were subjected to one-way ANOVA analysis in the Minitab statistical package (v.18.1). Data were analyzed in a completely randomized design and the analyzed data are presented as means ± SEM and probability values. Values were considered statistically different at *p* ≤ 0.05.

For the RNA-Seq analysis, adaptor sequences and low-quality scores containing bases (Phred score < 30) were trimmed from reads using Trimmomatic [55]. The resulting reads were aligned to the GRCg6 genome using STAR [56]. Read counts were obtained using HTSeq [57]. The R package DESeq2 [58] was used to identify differentially expressed genes between the groups. Nominal *p*-values were corrected for multiple testing using the Benjamini–Hochberg method. Gene ontology (GO) enrichment analysis was performed using the R package GOSeq [59]. Kyoto Encyclopedia of Genes and Genome (KEGG) Pathway Enrichment analyses of differentially expressed genes were performed on the PANTHER platform (http://pantherdb.org, accessed on 26 September 2021) [60].

## 3. Results

The 16S rRNA V4–V5 sequencing resulted in 8,774,523 quality read counts at an average of 60,934 counts per sample after quality filtering and demultiplexing. A total of 554 operational taxonomic units (OTUs) at the 97% sequence similarity level were obtained from all samples.

### 3.1. Microbiota Diversity

Internal sample α-diversity was estimated using the number of observed features (richness) and Shannon’s index (diversity). Rarefaction curves of observed features and Shannon’s index values reached a plateau in all samples, demonstrating that sequencing depth was adequate to cover the bacterial diversity in both ceca and ileal samples (Supplementary Figures S1 and S2).

**Figure 2:**
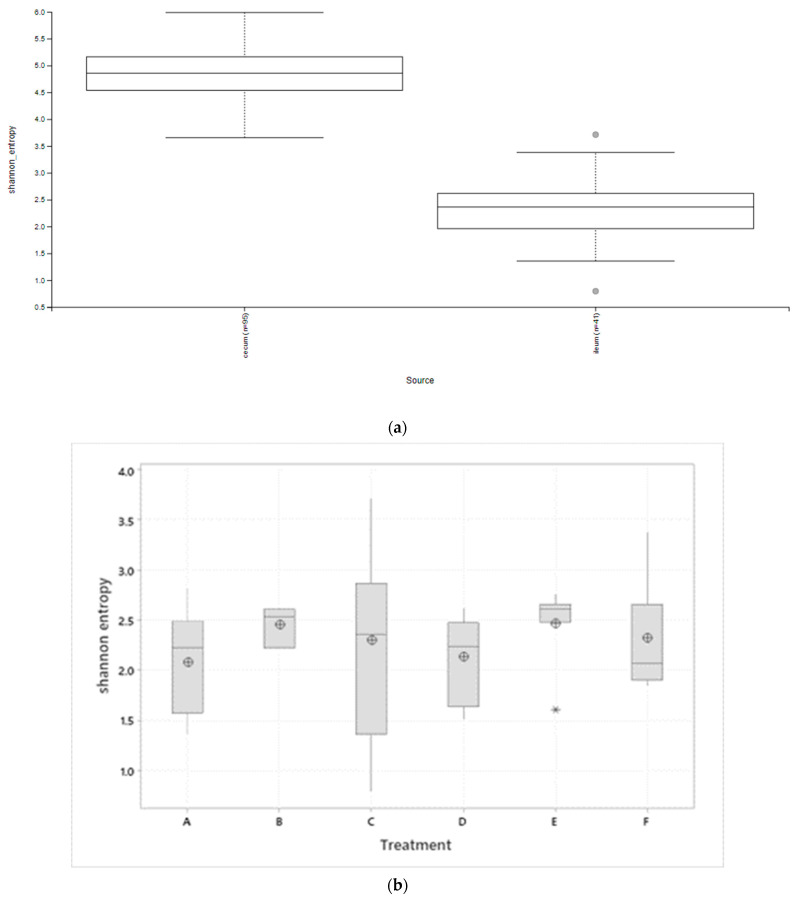

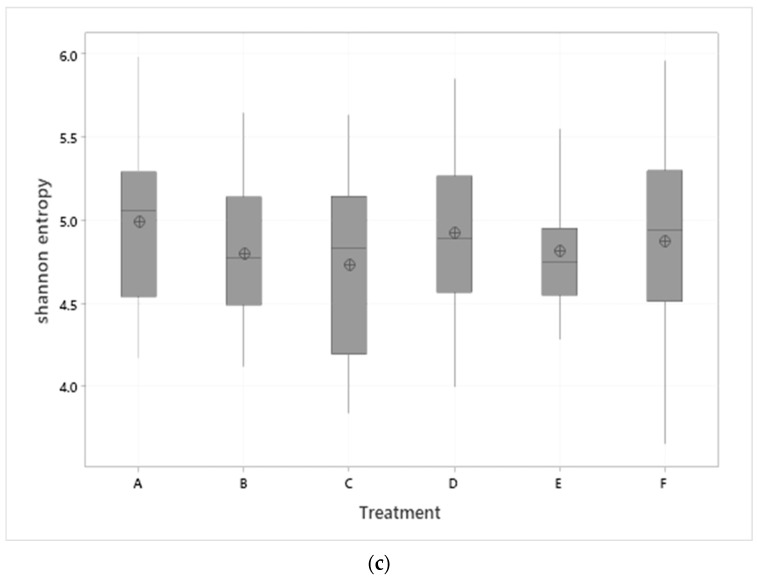
Alpha diversity (Shannon’s index) box plots show (**a**) significant difference between ileal and ceca microbiota (Kruskal–Wallis, *p* < 0.001), (**b**) no significant effect of treatments on ileal microbiota (Kruskal–Wallis, *p* > 0.05), (**c**) no significant effect of treatment on ceca microbiota (Kruskal–Wallis, *p* > 0.05). Treatments include (A) negative control treatment—chicks fed a basal corn-soybean meal-wheat-based diet; (B) in-feed antibiotics—chicks fed NC + 0.05% bacitracin methylene disalicylate and (C) in-water essential oil—chicks supplied the essential oil via the water route at the recommended dosage of 250 mL/1000 L of drinking water; (D) in ovo saline treatment—eggs injected with 0.2 mL of physiological saline (0.9% NaCl); (E) in ovo essential oil treatment—eggs injected with 0.2 mL of a saline + essential oil blend mixture at a dilution ratio of 2:1; and (F) in ovo + in-water essential oil treatment—chicks offered the essential oil blend via the in ovo and in water route, successively. Boxes in the boxplots denote interquartile range, solid middle line in the boxes denote the median, and ⊕ denote the means, Symbols ○ and * in (**a**,**b**) represent outliers.

Alpha diversity inspection revealed significant (*p* < 0.001) diversity between the ileal and ceca samples but not between treatment groups (Figure 2a–c). Ceca samples recorded a higher Shannon diversity index compared to ileal samples. While Shannon’s diversity index showed a similar profile between the treatment groups in both ceca and ileal tissues, the ovo EO treatment recorded numerically higher alpha diversity in the ileum, the same as the NC treatment in the ceca.

To determine beta diversity, a principal coordinate analysis (PCoA) based on unweighted UniFrac distances was conducted. The PCoA plot showed unique cluster separation between the ileal and ceca microbiota; contrastingly, no difference in microbial community structure between treatments in both the ileum and ceca was observed (Supplementary Figure S3).

### 3.2. Microbiota Composition

**Figure 3:**
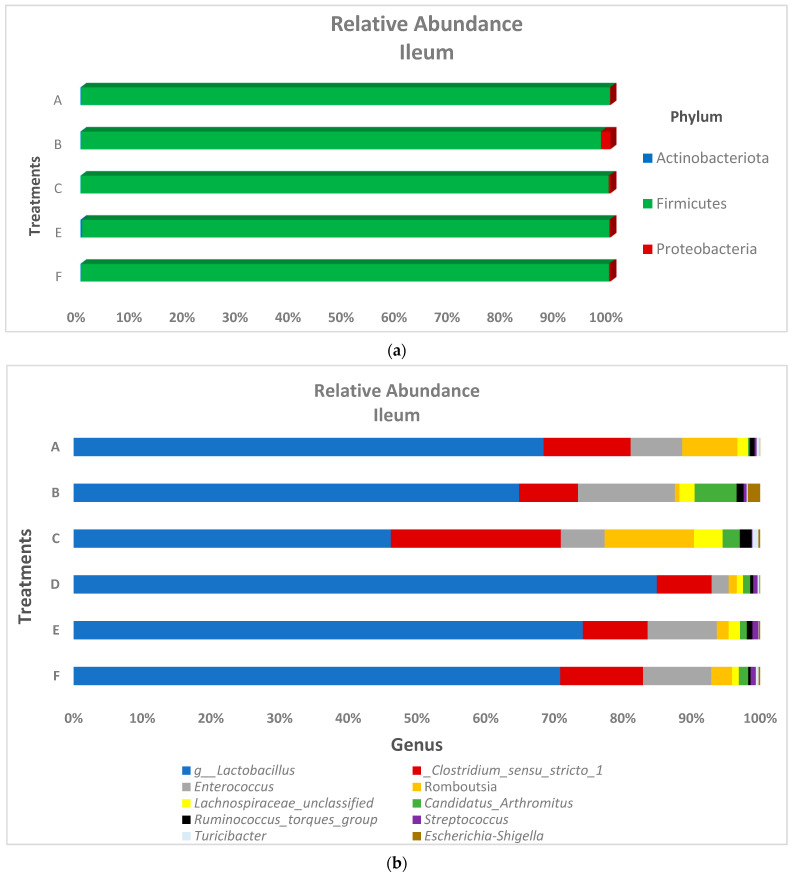
Ileal microbiota bacteria composition at the (**a**) phylum and (**b**) genus levels of broiler chickens subjected to different treatments groups. Treatments include (A) negative control treatment—chicks fed a basal corn-soybean meal-wheat-based diet; (B) in-feed antibiotics—chicks fed NC + 0.05% bacitracin methylene disalicylate; (C) in-water essential oil—chicks supplied the essential oil via the water route at the recommended dosage of 250 mL/1000 L of drinking water; (D) in ovo saline treatment—eggs injected with 0.2 mL of physiological saline (0.9% NaCl); (E) in ovo essential oil treatment—eggs injected with 0.2 mL of a saline + essential oil blend mixture at a dilution ratio of 2:1l; and (F) in ovo + in-water essential oil treatment—chicks offered the essential oil blend via the in ovo and in water route, successively.

**Figure 4:**
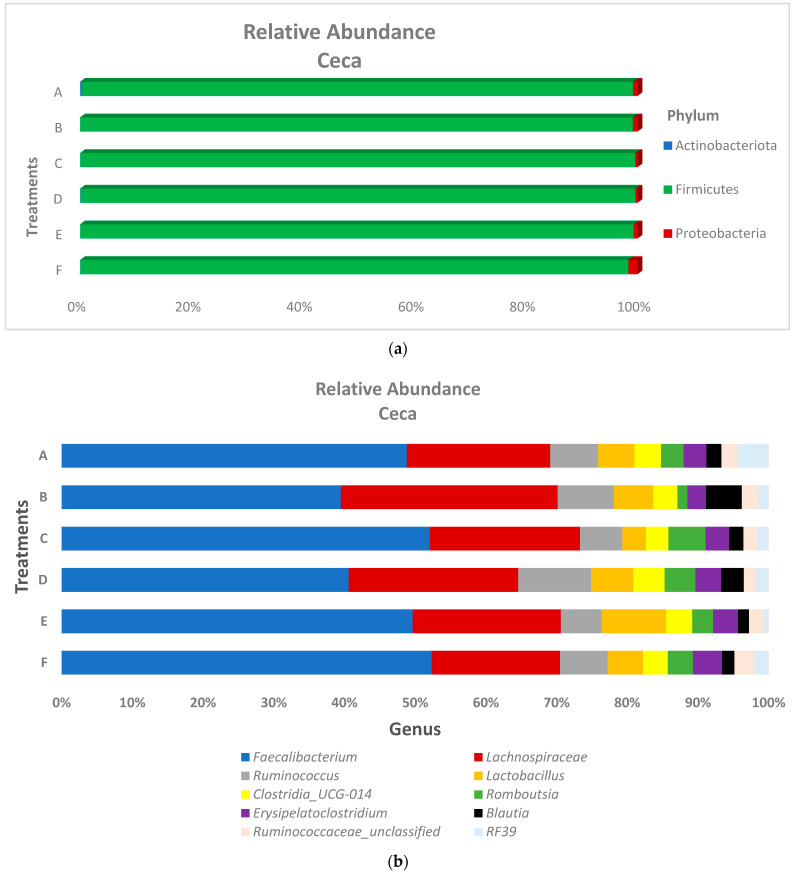
Ceca microbiota bacteria composition at the (**a**) phylum and (**b**) genus levels of broiler chickens subjected to different treatments groups. Treatments include (A) negative control treatment—chicks fed a basal corn-soybean meal-wheat-based diet; (B) in-feed antibiotics—chicks fed NC + 0.05% bacitracin methylene disalicylate; (C) in-water essential oil—chicks supplied the essential oil via the water route at the recommended dosage of 250 mL/1000 L of drinking water; (D) in ovo saline treatment—eggs injected with 0.2 mL of physiological saline (0.9% NaCl); (E) in ovo essential oil treatment—eggs injected with 0.2 mL of a saline + essential oil blend mixture at a dilution ratio of 2:1; and (F) in ovo + in-water essential oil treatment—chicks offered the essential oil blend via the in ovo and in water route, successively.

**Figure 5:**
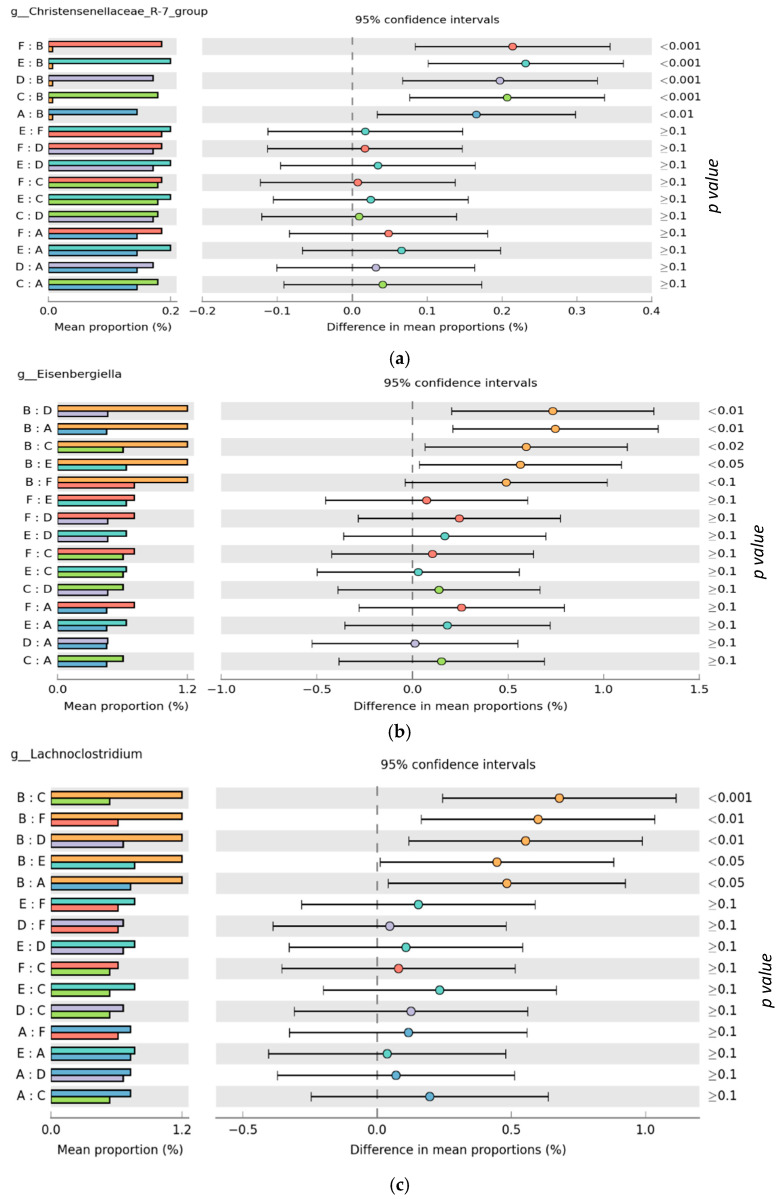

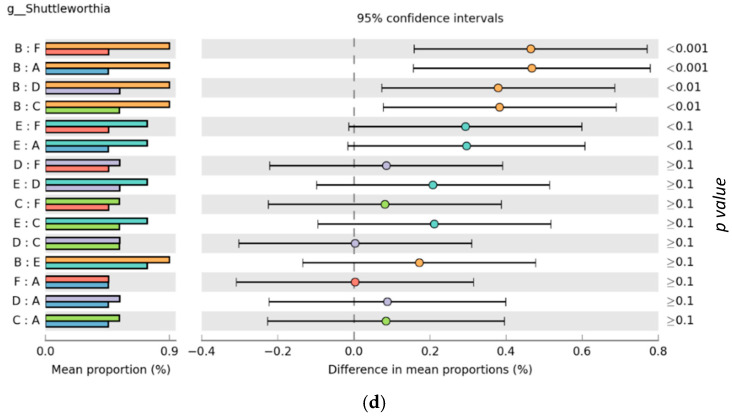
Significant differences (ANOVA, B–H FDR corrected *p* value: *p* < 0.05) in cumulative proportions of genus (**a**) *Christensenellaceae_R-7*_group, (**b**) *Elsenbergiella*, (**c**) *Lachnoclostridium*, and (**d**) *Shuttleworthia* in ceca microbiota of broiler chickens subjected to different treatments groups. Treatments include (A) negative control treatment—chicks fed a basal corn-soybean meal-wheat-based diet; (B) in-feed antibiotics—chicks fed NC + 0.05% bacitracin methylene disalicylate; (C) in-water essential oil—chicks supplied the essential oil via the water route at the recommended dosage of 250 mL/1000 L of drinking water; (D) in ovo saline treatment—eggs injected with 0.2 mL of physiological saline (0.9% NaCl); (E) in ovo essential oil treatment—eggs injected with 0.2 mL of a saline + essential oil blend mixture at a dilution ratio of 2:1; and (F) in ovo + in-water essential oil treatment—chicks offered the essential oil blend via the in ovo and in water route, successively.

The relative abundance of the predominant bacteria phyla and genus in both the ileum and ceca are presented in Figure 3 and Figure 4, respectively. At the phyla level, ileum microbiota was dominated by Firmicutes (range of 99.5–99.8%), Proteobacteria (range of 0.03–1.79%), and Actinobacteria (range of 0.03–0.12%) for all treatments. Ceca microbiota phyla taxa showed a similar trend as the ileal microbiota, as the relative abundance of Firmicutes (range of 98.3–99.6%) was found higher than Proteobacteria (range of 0.38–0.81%), which was also higher than Actinobacteria (range of 0.01–0.24%) across the treatment groups. At the genus taxa, the ileal microbiota was 96% dominated by *Lactobacillus*, *Clostridium sensu_stricto_1*, *Enterococcus*, *Romboutsia*, and *Lachnospiraceae_unclassified* species, with *Lactobacillus* species being the prevalent species (occurring > 64% in all treatments, except for the in-water EO treatment, which recorded a 46.2% *Lactobacillus* relative abundance). *Faecalibacterium* was the most abundant genus in the ceca, recording at least 40% abundance across treatment groups. Similar to the ileal microbiota, genus *Lactobacillus* and *Romboutsia* were also found in the ceca, although at lower relative abundance. Contrastingly, the genus *Lachnospiraceae* was higher in the ceca (22.39%) compared to the ileum (1.91%). Significant differences in the cumulative proportions of bacteria in the genera *Christensenellaceae_R-7_group*, *Elsenbergiella*, *Lachnoclostridium*, and *Shuttleworthia* were recorded between treatments in the ceca (Figure 5). Compared to other treatments, the in-feed antibiotic treatment significantly (*p* < 0.05) increased the proportion of *Eisenbergiella*, *Lachnoclostridium*, and *Shuttleworthia*. Contrastingly, the proportion of bacteria *Christensenellaceae_R-7_group* (*p* < 0.01) in the ceca was reduced by the in-feed antibiotic treatment when compared to other treatments. No significant differences in the microbiota proportion between treatments were recorded in the ileum at both the phylum and genus levels.

### 3.3. Ceca SCFA Concentration

The resulting concentrations of ceca SCFA are presented in Table 1. Only the concentration of butyric acid recorded a statistical trend towards significance (*p* = 0.09) in the in-water EO treatment, compared to other treatments. All other acids that were quantified recorded no statistical significance between treatment groups (*p* > 0.05). Nonetheless, the in-water essential oil treatment equally recorded numerically higher concentrations of acetic, lactic, volatile, and total fatty acids. Total bacteria (copies/gram of sample) were also found to be higher (*p* > 0.05) in the in-water EO treatment when compared to other treatments.

### 3.4. Transcriptome Analysis

In this study, to identify differentially expressed mRNAs in the liver of broiler chickens, a total of 6,360,427,350 raw reads were generated from 48 samples (Supplementary Table S2). After the trimming step, 6,357,176,660 clean reads were obtained and the clean reads were aligned to the whole genome of *Gallus gallus domesticus* (Fasta: *Gallus_gallus*. GRCg6a.fa, Annotation: Gallus_gallus.GRCg6a.Ensembl98.gtf, source: Ensembl98). An average of 95.56% of clean reads were mapped to the genome. To determine the statistical significance of differentially expressed genes (DEGs) between treatments, the genes with the parameter of false discovery rate (FDR < 0.05) were considered differentially expressed genes/transcripts. Only a limited number of DEGs were observed (6: 3 up-regulated, and 3 down-regulated) to be differentially influenced by the treatments (Table 2). The in ovo + in-water EO recorded the highest number (2) of DEGs in this category, as it down-regulated the expression of both cubilin (CUBN) and aldehyde dehydrogenase 1 family member L2 (ALDH1L2) genes. A heatmap illustrating the top 100 most variable genes is presented in Supplementary Figure S4.

## 4. Discussion

The supplementation of phytogenic feed additives, especially essential oil, is reported to promote lipid and cholesterol metabolism [39], as well as to enhance immunity [61], leading to improved poultry performance. These favorable effects are thought to be exerted through the modulation of gut microbiota and the expression of several unique genes [39,40,41,44,61]. However, in vivo results on the effect of EO on chicken microbiota are somewhat inconsistent. While EO blends have been reported to reduce the relative abundance of pathogenic bacteria like *Escherichia coli* [22,23], *Salmonella* [24], *and Clostridium perfringens* [25] in broiler chickens, a few studies have equally reported no effect of EO supplementation on gut commensal bacteria [24,26,27]. This study utilized 16S rRNA gene sequencing in combination with transcriptomic analysis to investigate the effect of essential oil and its delivery routes (in water, in ovo, and in ovo + in water) and 0.05% bacitracin on both the composition and diversity of ileal and ceca microbiota and liver transcriptomics. Additionally, ceca short-chain fatty acid concentration was also evaluated. Bacitracin, the positive control in this study, is an extensively used antibiotic growth promoter in the poultry industry [62]. There is no doubt that the detailed delineation of the effect of a classic AGP like bacitracin and an alternative to AGP and its delivery routes, as this study presents, is key to understanding the molecular mechanisms underlying growth promotion in poultry. Accordingly, this study provides insight into the microbiota-mediated mode of action of antibiotics growth promoters, as well as preliminary transcriptomic evidence suggesting sex-controlled hepatic differential gene expression in broiler chickens offered antibiotics and essential oil (via water, in ovo, and in ovo + in-water delivery routes).

The gut microbiota plays an important role in host health, immune modulation, nutrient absorption, and pathogen control [63,64]. Although no treatment effect was recorded, this study revealed higher alpha (Shannon index) diversity in broiler chicken ceca compared to the ileum. This agrees with other studies [65,66,67], which have also recorded higher microbial diversity in the ceca. Higher microbial richness and stability observed in the ceca compared to the ileum in broiler chicken have been correlated with the higher number of obligate anaerobic microbes present therein, compared to aerobes or facultative anaerobes [63]. Consistent with the results of this study, both Abdelli et al. [68] and Pham et al. [69] have equally reported no effect of EO on alpha diversity. Thibodeau et al. [70] have shown that only extreme events (dysbiosis and disease inclusive) which modify the number of ecological niches in different bacterial species can alter the alpha diversity. Furthermore, beta diversity analysis revealed no difference in microbial community structure between treatments at both the ileum and ceca; however, the bacteria communities clearly differed across both gut sections. Other studies involving AGP or EO supplementation in broiler chickens have also observed similar results [71,72,73]. Conversely, Pham et al. [69] have recently highlighted the potential of EO to modulate the gut bacterial community structure. Aside from differences in intestinal sections, time of sampling, and supplemented additives, other factors, including broiler chicken breed, age, environmental condition, and disease status, can potentially cause shifts in beta diversity [63,74]. Diseases accompanied by intestinal dysbiosis like necrotic enteritis and *Eimeria* infection are reported to cause a significant change in gut microbiota community structure [75,76], buttressing the healthy state of the flock in this study.

Furthermore, in-feed antibiotic treatment significantly (*p* < 0.05) increased the proportion of *Eisenbergiella*, *Lachnoclostridium*, and *Shuttleworthia*, while decreasing (*p* < 0.01) the proportion of *Christensenellaceae_R-7*_group in the ceca, as compared to other treatments in this study. Interestingly, all of the bacteria with an increased proportion belong to the family *Lachnospiraceae*. The abundance of bacteria in the family *Lachnospiraceae* has been associated with improved weight gain [77], feed conversion ratio [78], and butyrate production in broiler chickens [79,80]. The performance result from this study published in Oladokun et al. [47] shows the increased weight gain recorded by the in-feed antibiotic treatment in the early period (d 1–14), compared to the in ovo treatments group, supports this result. Consistent with our results, Zhong et al. [81] reported the increased abundance of bacteria in the genus *Eisenbergiella* in neonates offered probiotics and antibiotics concurrently. An increased abundance in bacteria of the genus *Eisenbergiella* has been associated with reduced incidence of gastrointestinal disorders linked to metabolic and microbiota changes (functional dyspepsia), resulting in improved nutrient metabolism in broiler chickens [82]. Nonetheless, a few studies have also associated the abundance of this genera with the incidences of subclinical enteritis and *Eimeria* infection in broiler chickens [83,84], emphasizing the cost-benefit effects of antibiotic use in poultry production and the need for more studies in this regard. *Lachnoclostridium* has been positively correlated with increased butyrate production with attendant gut health protection and pathogen control effects [85,86]. Probiotics [87], prebiotics (wheat bran) [88], and antibiotics, but not essential oil, have all been reported to enrich the abundance of *Lachnoclostridium* in broiler chicken ceca [89]. Similar to other enriched genera in the ceca in this study, the genus *Shuttleworthia* has also been associated with increased weight gain and growth performance resulting from a possible role in lipid and carbohydrate metabolic pathways [77]. Furthermore, disease conditions like avian leukosis virus [90], coccidia infection [91], and *Salmonella* infection have all been reported to decrease the abundance of bacteria in the genus *Shuttleworthia* in broiler chicken ceca [92]. Similar to the results presented here, Hung et al. [93] have also reported a reduced abundance of members of the genus *Christensenellaceae* in the feces of weaned piglets offered the antibiotic bacitracin. Although the functional role of bacteria in this genus in the chicken microbiota is not fully known, their abundance has been associated with the colonization *of Campylobacter jejuni*, a foodborne zoonotic pathogen [70]. While the results presented here suggest that antibiotic growth promoters might give the birds a better growth advantage than EOs under this experimental condition, such growth advantage might come with some metabolic costs, deducible from the metabolic functions of bacteria species enhanced by this treatment. Hence, more research is needed on potent alternatives to antibiotic growth promoters with no reported adverse effects on the poultry industry. Nonetheless, the results on gut microbiota presented here provide a critical perspective on microbiota-mediated mode of action of antibiotics growth promoters in broiler chickens.

The fermentation of dietary fibers to yield SCFA constitutes an important function of the ceca commensal microbiota. No significant effect of evaluated treatments on ceca SCFA concentration was recorded in this study. Only the in-water EO treatment showed a statistical trend (*p* = 0.09) of enhancing ceca butyric acid concentration relative to other treatments. Butyric acid serves as an important energy substrate for the maintenance and proliferation of gut colonic cells and structures [71,94]. Essential oils (in feed or in water) have been reported to increase the concentrations of acetic, butyric, propionic, and lactic acids and total SCFA in quail breeders [95] and broiler chickens [96,97,98]. The positive effect of EO on ceca SCFA concentration could be related to the capacity of their phytogenic formulations to enhance bacteria proliferation in the lower gut. Several variable factors that potentially influence SCFA concentrations in broiler chickens, including the microbiota composition, bird age, and the amount and type of available fermentable substrates, explain the observed results in this study [23,99,100].

Transcriptomic analysis in this study suggests unique sex-controlled gene expression in broiler chicken livers. To evaluate the similarities and dissimilarities between samples in an unsupervised manner, principal component exploratory analysis (PCA) was carried out. The PCA showed that samples were not segregated by treatments (Supplementary Figure S5) but instead showed modest segregation based on an unknown variable, probably sex. To confirm the hypothesis that treatments indeed clustered based on sex, the expression of five highly expressed genes on the W chromosome was examined. The results showed that all five genes showed much higher expression in group two than group one, indicating that group two samples were probably female (group two were samples with PC1 score > 0, group one were samples with PC1 scores < 0) (Supplementary Figure S6). Based on these PCA gene expression plots, samples were thus assigned as male or female according to their PC1 score. A total of 14 DEGs were found to be influenced by the treatment and sex (Supplementary Table S3). Of these DEGs, six genes were up-regulated (fold change range from 0.4 to 1.1) and eight genes were down-regulated (fold change range from −0.5 to −0.9). Sex-based analysis revealed that in male transcripts, antibiotic treatments recorded the highest number of DEGs (seven genes: four upregulated and three downregulated) compared to other treatments. Similarly, in male transcripts, the BVES (blood vessel epicardial substance) gene was significantly downregulated in both antibiotics, in-water essential oil, and in ovo essential oil treatments. In female birds, only four DEGS (two upregulated, two downregulated) were recorded amongst treatment groups. To understand the functional roles of identified DEGs, GO and KEGG pathway analysis was carried out. The GO analysis showed that a total of 33 significant GO categories were enriched (*p* < 0.05) compared to the negative control treatment (Supplementary Figure S7a,b). The GO terms included both biological process (BP), cellular component (CC), and molecular function (MF). However, a vast majority (75.8%) of the significant GO terms in the male transcript were observed in the antibiotic treatment, with GO terms in the BP category including “non-canonical Wnt signaling pathway” and “snRNA 3′-end processing” being the principal terms. In the female transcript, the vast majority (51.5%) of the significant GO terms were observed in the in-water EO treatment, with GO terms in the CC category including “signal transduction” and “plasma membrane” being the principal terms. The KEGG pathway analysis results, also shown in Table 2, provides predictions of differentially regulated pathways across treatments and sex. The results revealed that the main enriched pathways were cell signaling- (acting in sodium-glucose transporter, ion channels, exosome, and inositol phosphate metabolism) and tight-junction-related pathways. Other highlighted enriched pathways include genetic information processing, transcription machinery, spindle formation proteins, phosphoric diester hydrolases, and purine metabolism.

Several factors, including the threshold of significance levels, but certainly not the number of animals or sample number utilized in this study, could have contributed to the low number of DEGs observed in this study (n = 14). Compared to this study, other broiler chicken RNA-Seq experiments [101,102,103] have utilized lower animal or sample numbers to detect a higher number of DEGs. The BVES gene with the cellular GO term category “regulation of microtubule cytoskeleton organization and tight junction related pathways” was found to be ubiquitously downregulated in male transcripts in this study irrespective of treatments, suggesting that this gene plays a vital metabolic function in the cell. Since first identified in 2001, the BVES gene has mostly been functionally correlated with the maintenance of epithelial integrity and tight junctions [104,105,106,107]; this has been validated by decreased trans-epithelial resistance values (TER, a measure of tight junction integrity) [106]. Nonetheless, the exact mechanisms of its role in tight junction maintenance are yet to be fully elucidated [108]. Osler et al. [106] have proposed that BVES’s role in tight junction maintenance might indeed be a secondary effect, with more primary roles likely related to cell signaling, and structural support, among others. Besides the intestine, where tight junction proteins are noted to ensure gut barrier integrity, the BVES gene is also reported to be highly expressed in cardiac and skeletal muscle [109,110,111]. More recently, Gu et al. [112] reported the detection of BVES following whole-genome resequencing of the autochthonous Niya chicken breed and associated its function to the regulation of heart rate and heart development [113,114]. Considering that ischemic hepatic necrosis, which is linked to heart failure, could occur in broiler chickens [115] and the healthy state of flocks in this study, it is hypothesized that the downregulation of the BVES gene in broiler chicken liver observed in male transcripts in this study might be functionally related to the regulation of heart rate. Moreover, male embryos and adult chickens are reported to exhibit slower heart rates as compared to females [116,117]. More studies are thus needed to validate the relationship between BVES expression in the liver and heart rate regulation in broiler chickens.

Furthermore, antibiotic treatment upregulated the expression of INTS2 (integrator complex subunit 2), SLC5A10 (solute carrier family 5-member 10), CEP70 (centrosomal protein 70), and MED13 (mediator complex subunit 13) genes, while downregulating the expression of PDE11A (phosphodiesterase 11A) and CLCA1 (chloride channel accessory 1) genes in male transcripts in this study. Only in female transcripts did the antibiotic treatment upregulate PANX2 (pannexin 2) gene expression. INTS2 is a subunit of the integrator complex, which interacts with the C-terminal domain (CTD) of RNA polymerase II (RNAP II) large subunit and modulates 3-prime end processing of small nuclear RNAs (snRNAs) U1 and U2 [118]. The snRNAs are components of the spliceosome involved with the processing of pre-mRNA while also modulating the expression of other genes [119]. The modulation of snRNAs has also been reported to impact the innate immune system [120]. Slc5a10 (encoding SGLT5) is a mannose, fructose, and to a less degree, a glucose and galactose transporter [121,122]. Although glucose transporter 2 (GLUT2) is considered the main sugar transporter relevant to liver function [123], Fukuzawa et al. [124] have reported exacerbated hepatic steatosis induced by diminished sodium-dependent fructose uptake in SGLT5-deficient mice, suggesting the potential use of this gene as an indirect biomarker of liver health. Moreover, while the liver is the main site of ingested fructose metabolism, the occurrence of excess fructose beyond the liver’s metabolic capacity triggers GLUT5 transporter upregulation to ensure fructose absorption into the epithelial cells [125]. Similar to the BVES gene with predicted cardioprotective effect, the upregulation of the MED13 gene by the antibiotic treatment is also thought to exert a cardioprotective effect in the birds. The MED13 gene is a component of the mediator complex, working in synchronization with RNA polymerase II to direct transcription [126]. Its mutation has been implicated in lethal cardiac defects [127,128,129]. Similarly, upregulated CEP70 expression, as induced by antibiotic treatment in this study, has been implicated in the pathophysiology of numerous cancers [130]. It is a centrosomal-associated protein that has been linked with the regulation of microtubule nucleation in animal cells [131,132,133]. Centrosome dysfunction has been linked to the incidences of liver diseases and other non-apparent cell cycle defects in humans [134]. The downregulated PDE11A is a dual-specificity phosphodiesterase that catalyzes the breakdown of the cyclic nucleotides cyclic adenosine monophosphate (cAMP) and cyclic guanosine monophosphate (cGMP) [135]. Although mainly expressed in the prostate, it also finds expression to a lower degree in the pituitary gland, heart, and liver [136]. Although CLCA1 is noted for its role in the activation of calcium-activated chloride channels, its downregulation is reported to enhance pro-inflammatory cytokine release in both mice mucus cells [137] and human small airway epithelial cells [138]. Increased innate immune responses are usually associated with increased energy demands; this suggests that antibiotic use might be an energy-intensive means of growth promotion. Similar to this study, Farmahin et al. [139] have reported the differential expression of PANX2 in the liver of female, but not male, Fischer rats. PANX2 is functionally known for its potential to create gap junctions that facilitate ion exchange between cells and their role as a potential tumor suppressor in the human brain, skin, and liver tissues [140,141,142,143].

In a like manner, in-water EO treatment equally downregulated the expression of the MC5R (melanocortin 5 receptor) gene in female transcripts in this study. MC5R encodes a protein receptor for melanocyte-stimulating hormone and adrenocorticotropic hormone. It has been functionally designated as a candidate gene for obesity and fatness in humans and domestic animals [144]. Consistent with the results presented here, Ren et al. [145] have previously reported that its expression in the chicken liver might be estrogen activated, further buttressing its differential expression in female transcripts in this study. Similarly, Blankenship et al. [146] have reported that the downregulation of MC5R was critical to achieving feed efficiency phenotype in first-generation female, but not male, quails in their study. This is likely achieved directly by fatty acid metabolism or indirectly by glucose homeostasis. This result is not unexpected, considering that the broiler chickens utilized in this study have been bred for high feed efficiency. Conversely, the in-water EO treatment up-regulated the expression of the GUCY2C (guanylate cyclase 2C) gene, which encodes guanylate cyclase belonging to the membrane guanylyl cyclase family [147]. Mice deficient in GUCY2C have been reported to have reduced inflammatory response due to reduced expression of pro-inflammatory molecules [148]. In contrast, higher expression of GUCY2C in the liver of milk-restricted lambs has been associated with increased pro-inflammatory response [149]. This is likely the molecular basis of the antibacterial properties of essential oils, especially as it relates to pro-inflammatory hepatic stimulus. This also has an energy trade-off, as more energy might be directed towards countering systemic inflammation and not growth. Higher expression of GUCY2C in human females as compared to males has also been reported [150]. Furthermore, while the in ovo EO treatment downregulated the expression of the cubilin (CUBN) gene, the in ovo + in-water EO treatment downregulated the expression of the MTMR6 (myotubularin related protein 6) gene in male and female transcripts, respectively. Although the functional relevance of CUBN in chickens is not fully understood, CUBN is generally noted to play a role in the uptake of vitamin, iron, and lipoprotein endocytosis [151,152]. Lee et al. [153] have reported the downregulation of the CUBN gene in chicken lines with high residual feed intake, suggesting a possible role in amino acid metabolism and molecular transport network. Sun et al. [154] have also alleged that the downregulation of this gene could be induced by stressors, particularly heat stress. More research is thus needed to fully elucidate the functionality of this gene in chickens. Overexpression of the downregulated MTMR6 by the in ovo + in-water EO treatment has been reported to inhibit the Ca^2+^-activated potassium channel [155,156]. Given the physiological function of the Ca^2+^-activated potassium channel, which is to regulate cellular membrane potential and calcium signaling, this is considered to be a beneficial effect of essential oil delivery via this route. Moreover, hypoxia has been reported to increase mitoBKCa channel activity (big conductance potassium channel) of rat liver [157].

Furthermore, in this study, both in-feed antibiotic and in ovo + in-water EO treatments downregulated the expression of ALDH1L2 in the liver of broiler chickens. Similar to this study, Li et al. [158] have previously reported the downregulation of ALDH1L2 in a nonalcoholic steatohepatitis (NASH) rat model by analyzing the liver proteome, suggesting that ALDH1L2 may be involved in NASH progression. Contrary to the result presented here, Bajagai et al. [101] have reported upregulation of the ALDH1L2 gene with continuous EO (2% oregano powder) supplementation in the liver of male broiler chickens. Differences in routes of EO supplementation and length of study may have possibly influenced reported results. Although little is known about the BTN1A1 gene in chickens, which was upregulated by the in-water EO treatment in this study, Huang et al. [159] reported that this gene might play a role in immune response via inhibition of T-cell activation using lipopolysaccharide-challenged broiler chickens [160,161]. Low hepatic expression of this gene has also been reported in water buffalo [162]. In addition, while the ST8SIA6 gene has been reported to be downregulated in the liver of apolipoprotein E-knockout (apo E-KO) mice offered phytosterol treatment for 14 weeks [163], an upregulation of this gene in birds of the in ovo EO treatment was observed in this study. Phytosterols are of plant origin and have reported cholesterol-lowering effects [164,165]. In humans, high expression of the ST8SIA6 gene has been attributed an oncogenic function, including tumor cell proliferation, invasion, and migration [166]. As little is known of this gene in chickens, an overt attribution of a high expression of this gene to an increased likelihood of hepatic steatosis or liver cancer might be farfetched. More studies are needed in this regard to enable a more definite prognosis. On the other hand, the CUBN gene is reported to play an important role in the metabolism and transport of the active form of vitamin D (1,25-dihydroxy vitamin D) in the liver. This has been confirmed in transcriptomics studies involving mice supplemented with cholecalciferol [167,168]. Although this gene is reported to be downregulated by the in ovo EO treatment in this study, Collision et al. [169] have previously reported its upregulation in mice liver under a trans-fatty acid (TFA)-induced non-alcoholic fatty liver disease challenge. Overall, the results presented here provide transcriptomic evidence on the possibility of “natural” phytobiotics (including essential oil) having side effects depending on the length of use, dosage, and administration routes, an important concept to be considered in the development of potent human and animal pharmacotherapeutic strategies.

## 5. Conclusions

Summarily, while treatments yielded no difference in alpha and beta bacteria diversity in this study, clear differences in ileal and ceca microbiota distribution and structure were recorded. In-feed antibiotic treatment is also reported to significantly increase the proportion of specific beneficial bacteria in the family *Lachnospiraceae* while reducing the proportion of bacteria in the genus *Christensenellaceae*, all in the ceca. No significant effect of the evaluated treatments on ceca SCFA concentration was recorded in this study. Only the concentration of butyric acid recorded a statistical trend towards significance in the in-water essential oil treatment when compared to other treatments. The study also suggests unique sex-controlled gene expression in broiler chicken liver. Compared to the negative control treatment, the differential expression of the INTS2, SLC5A10, MED13, CEP70, PDE11A, and CLCA1 genes functionally associated with genetic information processing, glucose transport, mediator complex, spindle formation proteins, phosphoric-diester hydrolases, and ion channel activity, respectively, were all regulated by the antibiotic treatment in male transcripts. Only the BVES and CUBN gene sets, functionally associated with tight junctions and cholesterol homeostasis, were regulated by the in-water and in ovo EO treatments in male transcripts, respectively, compared to the negative control treatment. Conversely, in female transcripts, while the antibiotic treatment regulated the expression of the PANX2 gene functionally associated with ion exchange, the in-water and in ovo + in-water treatments regulated the differential expression of GUCY2C, MC5R, and MTMR6 genes functionally associated with peptide hormone binding, melanocortin receptor activity, and peptidyl-tyrosine dephosphorylation, respectively, all compared to the negative control treatment. Taken together, the results presented here provide mechanistic insights on the microbiota-mediated mode of action of antibiotics growth promoters by modulating the abundance of specific bacteria communities, as well as preliminary transcriptomic evidence suggesting sex-controlled hepatic differential gene expression in broiler chickens offered antibiotics and essential oil (via water, in ovo, and in ovo + in-water delivery routes). To our knowledge, this is the first study to suggest such sex-controlled hepatic differential gene expression in broiler chickens offered these treatments. There is thus a need for well-designed in vivo studies that take sex into consideration in order to fully validate the results presented herein. Nonetheless, the data presented here not only provide guidance on antibiotics and essential oil application in the poultry industry; they also provide a solid framework for further research in the field.

## Figures and Tables

**Table 1:** Effect of essential oil delivery route on ceca short-chain fatty acid concentration (SCFA) and total eubacteria (copies/gram of sample) in broiler chickens.

Short-Chain Fatty Acid Concentration (mmol/kg)	Treatments ^1^						SEM ^2^ *p* Value	
	Negative Control	In-Feed Antibiotics	In-Water Essential Oil	In Ovo Saline	In Ovo Essential Oil	In Ovo Essential Oil + In-Water Essential Oil		
Acetic acid	51.9	50.5	57.2	55.4	50.9	55.8	1.73	0.82
Propionic acid	4.24	3.79	4.31	4.43	3.81	4.36	0.18	0.86
Butyric acid	13.6	15.7	19.3	18.8	13.4	17.9	0.77	0.09
Valeric acid	1.05	0.79	0.87	1.17	1.08	1.07	0.07	0.67
Lactic acid	0.60	0.73	1.40	1.26	1.09	0.83	0.86	0.49
Total SCFA	74.1	74.2	89.7	84.1	74.4	82.4	2.65	0.41
Branched-chain fatty acids	2.25	1.63	1.94	2.28	1.77	1.91	0.11	0.46
Volatile fatty acids	73.1	72.4	83.6	82.1	70.9	81.0	2.40	0.49
Total eubacteria (copies/gram of sample)	2.3 × 10^12^	1.9 × 10^12^	3.0 × 10^12^	2.6 × 10^12^	2.5 × 10^12^	2.2 × 10^12^	2.06 × 10^12^	0.72

^1^ Treatments include (1) negative control treatment—chicks fed a basal corn-soybean meal-wheat-based diet; (2) in-feed antibiotics—chicks fed NC + 0.05% bacitracin methylene disalicylate; (3) in-water essential oil-chicks supplied the essential oil via the water route at the recommended dosage of 250 mL/1000 L of drinking water; (4) in ovo saline treatment—eggs injected with 0.2 mL of physiological saline (0.9% NaCl); (5) in ovo essential oil treatment—eggs injected with 0.2 mL of a saline + essential oil blend mixture at a dilution ratio of 2:1; (6) in ovo + in-water essential oil treatment—chicks offered the essential oil blend via the in ovo and in water route, successively. ^2^ SEM = pooled standard error of means. Mean values from n = 16 birds/treatment group are presented.

**Table 2:** Differentially expressed genes in the liver of broiler chickens as influenced by treatment groups ^1^.

Treatments	Gene Symbol	Gene Description	Expression Level	log2FoldChange	*p* Value
B vs. A	ALDH1L2	aldehyde dehydrogenase 1 family member L2	Down	−0.6	<0.01
C vs. A	BTN1A1	butyrophilin subfamily 1 member A1-like	Up	0.4	0.02
D vs. A	AVD	Avidin	Up	0.5	<0.01
E vs. A	ST8SIA6	ST8 alpha-N-acetyl-neuraminide alpha-2,8-sialyltransferase 6	Up	0.6	<0.01
	CUBN	Cubilin	Down	−0.7	0.01
F vs. A	ALDH1L2	aldehyde dehydrogenase 1 family member L2	Down	−0.6	0.01

^1^ Treatments include (A) negative control treatment—chicks fed a basal corn-soybean meal-wheat-based diet; (B) in-feed antibiotics-chicks fed NC + 0.05% bacitracin methylene disalicylate; (C) in-water essential oil—chicks supplied the essential oil via the water route at the recommended dosage of 250 mL/1000 L of drinking water; (D) in ovo saline treatment—eggs injected with 0.2 mL of physiological saline (0.9% NaCl); (E) in ovo essential oil treatment—eggs injected with 0.2 mL of a saline + essential oil blend mixture at a dilution ratio of 2:1; (F) in ovo + in-water essential oil treatment-chicks offered the essential oil blend via the in ovo and in water route, successively. Each comparison is specified in the format “B vs. A”, where group B is compared to group A, with group A being the denominator for the comparison. Liver tissues (50–100 mg) were sampled from 8 replicate birds/treatment (independent of sex) using 1 mL TRIzol™ (Qiagen, Hilden, Germany).

## Data Availability

All data relevant to this manuscript are available upon request from the corresponding author.

## Supplementary Materials

The following supporting information can be downloaded at: https://www.mdpi.com/article/10.3390/microorganisms10050861/s1, Figure S1: Rarefaction curves of observed features obtained from 16S rRNA gene V4–V5 sequences on the basis of (a) Microbiota source- cecum and ileum and (b) Treatment; Figure S2: Rarefaction curves of Shannon’s index obtained from 16S rRNA gene V4–V5 sequences on the basis of (a) Microbiota source- cecum and ileum and (b) Treatments; Figure S3: PCoA plots based on unweighted UniFrac metric; Figure S4: Heatmaps of the top 100 most variable genes; Figure S5: Principal component analysis (PCA) plot; Figure S6: Gene expression of the chrW gene; Figure S7: Gene ontology (GO) classifications of differentially expressed genes (DEGs) between treatment groups in (a) male and (b) female transcripts; Table S1: Ingredients, calculated, and analyzed compositions of experimental diets; Table S2: Sequencing data quality control metrics; Table S3: Differentially expressed genes in the liver of broiler chickens as influenced by sex and treatment groups.

## References

[1] Mahmood T., Guo Y. (2020). Dietary fiber and chicken microbiome interaction: Where will it lead to?. Anim. Nutr..

[2] Ma F., Xu S., Tang Z., Li Z., Zhang L. (2021). Use of antimicrobials in food animals and impact of transmission of antimicrobial resistance on humans. Biosaf. Health.

[3] Thakur A., Kumar A., Sharma M., Kumar R., Vanita B. (2019). Strategies to Minimize the Impact of Antibiotic Resistance in Livestock Production System. Int. J. Curr. Microbiol. Appl. Sci..

[4] Castanon J.I.R. (2007). History of the Use of Antibiotic as Growth Promoters in European Poultry Feeds. Poult. Sci..

[5] FDA (2013). Guidance for Industry #213-New Animal Drugs and New Animal Drug Combination Products Administered in or on Medicated Feed or Drinking Water of Food-Producing Animals: Recommendations for Drug Sponsors for Voluntarily Aligning Product Use Conditions with GFI# 209.

[6] Chicken Farmers of Canada Antibiotics. https://www.chickenfarmers.ca/antibiotics/..

[7] Paiva D., McElroy A. (2014). Necrotic enteritis: Applications for the poultry industry. J. Appl. Poult. Res..

[8] Rinttilä T., Apajalahti J. (2013). Intestinal microbiota and metabolites—Implications for broiler chicken health and performance. J. Appl. Poult. Res..

[9] Yadav S., Jha R. (2019). Strategies to modulate the intestinal microbiota and their effects on nutrient utilization, performance, and health of poultry. J. Anim. Sci. Biotechnol..

[10] Rowland I., Gibson G., Heinken A., Scott K., Swann J., Thiele I., Tuohy K. (2018). Gut microbiota functions: Metabolism of nutrients and other food components. Eur. J. Nutr..

[11] Burkholder K.M., Thompson K.L., Einstein M.E., Applegate T., Patterson J.A. (2008). Influence of Stressors on Normal Intestinal Microbiota, Intestinal Morphology, and Susceptibility to Salmonella Enteritidis Colonization in Broilers. Poult. Sci..

[12] Shi D., Bai L., Qu Q., Zhou S., Yang M., Guo S., Li Q., Liu C. (2019). Impact of gut microbiota structure in heat-stressed broilers. Poult. Sci..

[13] Kers J.G., Velkers F.C., Fischer E.A.J., Hermes G.D.A., Stegeman J.A., Smidt H. (2018). Host and Environmental Factors Affecting the Intestinal Microbiota in Chickens. Front. Microbiol..

[14] Lu J., Idris U., Harmon B., Hofacre C., Maurer J.J., Lee M.D. (2003). Diversity and Succession of the Intestinal Bacterial Community of the Maturing Broiler Chicken. Appl. Environ. Microbiol..

[15] Oviedo-Rondón E.O., Hume M.E., Barbosa N.A., Sakomura N.K., Weber G., Wilson J.W. (2010). Ileal and Caecal Microbial Populations in Broilers Given Specific Essential Oil Blends and Probiotics in two Consecutive Grow-Outs. Avian Biol. Res..

[16] Thompson K., Burkholder K., Patterson J., Applegate T.J. (2008). Microbial Ecology Shifts in the Ileum of Broilers During Feed Withdrawal and Dietary Manipulations. Poult. Sci..

[17] Shang Y., Kumar S., Oakley B., Kim W.K. (2018). Chicken Gut Microbiota: Importance and Detection Technology. Front. Veter. Sci..

[18] Bassolé I.H.N., Juliani H.R. (2012). Essential Oils in Combination and Their Antimicrobial Properties. Molecules.

[19] Gopi M., Karthik K., Manjunathachar H.V., Tamilmahan P., Kesavan M., Dashprakash M., Balaraju B.L., Purushothaman M.R. (2014). Essential oils as a feed additive in poultry nutrition. Adv. Anim. Vet. Sci..

[20] Stevanović Z.D., Bošnjak-Neumüller J., Pajić-Lijaković I., Raj J., Vasiljević M. (2018). Essential Oils as Feed Additives—Future Perspectives. Molecules.

[21] Swamy M.K., Akhtar M.S., Sinniah U.R. (2016). Antimicrobial properties of plant essential oils against human pathogens and their mode of action: An updated review. Evid.-Based Complement. Altern. Med..

[22] Cho J., Kim H., Kim I. (2014). Effects of phytogenic feed additive on growth performance, digestibility, blood metabolites, intestinal microbiota, meat color and relative organ weight after oral challenge with Clostridium perfringens in broilers. Livest. Sci..

[23] Hashemipour H., Khaksar V., Rubio L., Veldkamp T., van Krimpen M. (2016). Effect of feed supplementation with a thymol plus carvacrol mixture, in combination or not with an NSP-degrading enzyme, on productive and physiological parameters of broilers fed on wheat-based diets. Anim. Feed Sci. Technol..

[24] Pathak M., Mandal G.P., Patra A.K., Samanta I., Pradhan S., Haldar S. (2017). Effects of dietary supplementation of cinnamaldehyde and formic acid on growth performance, intestinal microbiota and immune response in broiler chickens. Anim. Prod. Sci..

[25] Mitsch P., Zitterl-Eglseer K., Köhler B., Gabler C., Losa R., Zimpernik I. (2004). The effect of two different blends of essential oil components on the proliferation of Clostridium perfringens in the intestines of broiler chickens. Poult. Sci..

[26] Hong J.-C., Steiner T., Aufy A., Lien T.-F. (2012). Effects of supplemental essential oil on growth performance, lipid metabolites and immunity, intestinal characteristics, microbiota and carcass traits in broilers. Livest. Sci..

[27] Paraskeuas V., Mountzouris K.C. (2019). Broiler gut microbiota and expressions of gut barrier genes affected by cereal type and phytogenic inclusion. Anim. Nutr..

[28] Bilia A.R., Guccione C., Isacchi B., Righeschi C., Firenzuoli F., Bergonzi M.C. (2014). Essential Oils Loaded in Nanosystems: A Developing Strategy for a Successful Therapeutic Approach. Evid.-Based Complement. Altern. Med..

[29] Heydarian M., Ebrahimnezhad Y., Meimandipour A., Hosseini S.A., Banabazi M.H. (2020). Effects of Dietary Inclusion of the Encapsulated Thyme and Oregano Essential Oils Mixture and Probiotic on Growth Performance, Immune Response and Intestinal Morphology of Broiler Chickens. Poult. Sci. J..

[30] Maenner K., Vahjen W., Simon O. (2011). Studies on the effects of essential-oil-based feed additives on performance, ileal nutrient digestibility, and selected bacterial groups in the gastrointestinal tract of piglets1. J. Anim. Sci..

[31] Malayoğlu H.B., Baysal Ş., Misirlioğlu Z., Polat M., Yilmaz H., Turan N. (2010). Effects of oregano essential oil with or without feed enzymes on growth performance, digestive enzyme, nutrient digestibility, lipid metabolism and immune response of broilers fed on wheat–soybean meal diets. Br. Poult. Sci..

[32] Mountzouris K., Paraskevas V., Tsirtsikos P., Palamidi I., Steiner T., Schatzmayr G., Fegeros K. (2011). Assessment of a phytogenic feed additive effect on broiler growth performance, nutrient digestibility and caecal microflora composition. Anim. Feed Sci. Technol..

[33] Oladokun S., Adewole D.I. (2020). In ovo delivery of bioactive substances: An alternative to the use of antibiotic growth promoters in poultry production—A review. J. Appl. Poult. Res..

[34] Slawinska A., Plowiec A., Siwek M., Jaroszewski M., Bednarczyk M. (2016). Long-Term Transcriptomic Effects of Prebiotics and Synbiotics Delivered In Ovo in Broiler Chickens. PLoS ONE.

[35] Roto S.M., Kwon Y.M., Ricke S.C. (2016). Applications of In Ovo Technique for the Optimal Development of the Gastrointestinal Tract and the Potential Influence on the Establishment of Its Microbiome in Poultry. Front. Veter. Sci..

[36] Glendinning L., Watson K.A., Watson M. (2019). Development of the duodenal, ileal, jejunal and caecal microbiota in chickens. Anim. Microbiome.

[37] Gong J., Si W., Forster R.J., Huang R., Yu H., Yin Y., Yang C., Han Y. (2007). 16S rRNA gene-based analysis of mucosa-associated bacterial community and phylogeny in the chicken gastrointestinal tracts: From crops to ceca. FEMS Microbiol. Ecol..

[38] Gong J., Chambers J.R., Wheatcroft R., Chen S., Forster R.J., Yu H., Sabour P.M. (2002). Molecular analysis of bacterial populations in the ileum of broiler chickens and comparison with bacteria in the cecum. FEMS Microbiol. Ecol..

[39] Sabino M., Capomaccio S., Cappelli K., Verini-Supplizi A., Bomba L., Marsan P.A., Cobellis G., Olivieri O., Pieramati C., Trabalza-Marinucci M. (2018). Oregano dietary supplementation modifies the liver transcriptome profile in broilers: RNASeq analysis. Res. Veter. Sci..

[40] Li W., He Z.-Q., Zhang X.-Y., Chen Y.-J., Zuo J.-J., Cao Y. (2020). Proteome and Transcriptome Analysis of the Antioxidant Mechanism in Chicken Regulated by Eucalyptus Leaf Polyphenols Extract. Oxidative Med. Cell. Longev..

[41] Bastos M.S., Del Vesco A.P., Santana T.P., Santos T.S., De Oliveira Junior G.M., Fernandes R.P.M., Barbosa L.T., Gasparino E. (2017). The role of cinnamon as a modulator of the expression of genes related to antioxidant activity and lipid metabolism of laying quails. PLoS ONE.

[42] Li H., Wang T., Xu C., Wang D., Ren J., Li Y., Tian Y., Wang Y., Jiao Y., Kang X. (2015). Transcriptome profile of liver at different physiological stages reveals potential mode for lipid metabolism in laying hens. BMC Genom..

[43] Akbarian A., Golian A., Gilani A., Kermanshahi H., Zhaleh S., Akhavan A., De Smet S., Michiels J. (2013). Effect of feeding citrus peel extracts on growth performance, serum components, and intestinal morphology of broilers exposed to high ambient temperature during the finisher phase. Livest. Sci..

[44] Lillehoj H.S., Kim D.K., Bravo D.M., Lee S.H. (2011). Effects of dietary plant-derived phytonutrients on the genome-wide profiles and coccidiosis resistance in the broiler chickens. BMC Proc..

[45] Rowsell H.C. Canadian Council on Animal Care: Its Role. https://www.ccac.ca/en/CCAC_Programs/Guidelines_Policies/GUIDES/ENGLISH/toc_v1.htm.

[46] Oladokun S., Koehler A., MacIsaac J., Ibeagha-Awemu E.M., Adewole D.I. (2021). Bacillus subtilis delivery route: Effect on growth performance, intestinal morphology, cecal short-chain fatty acid concentration, and cecal microbiota in broiler chickens. Poult. Sci..

[47] Oladokun S., MacIsaac J., Rathgeber B., Adewole D. (2021). Essential Oil Delivery Route: Effect on Broiler Chicken’s Growth Performance, Blood Biochemistry, Intestinal Morphology, Immune, and Antioxidant Status. Animals.

[48] National Research Council (1994). Nutrient Requirements of Poultry.

[49] Comeau A.M., Douglas G.M., Langille M.G.I. (2017). Microbiome Helper: A Custom and Streamlined Workflow for Microbiome Research. mSystems.

[50] Martin M. (2011). Cutadapt removes adapter sequences from high-throughput sequencing reads. EMBnet J..

[51] Bolyen E., Rideout J.R., Dillon M.R., Bokulich N.A., Abnet C.C., Al-Ghalith G.A., Alexander H., Alm E.J., Arumugam M., Asnicar F. (2019). Reproducible, interactive, scalable and extensible microbiome data science using QIIME 2. Nat. Biotechnol..

[52] Rognes T., Flouri T., Nichols B., Quince C., Mahé F. (2016). VSEARCH: A versatile open source tool for metagenomics. PeerJ.

[53] Amir A., McDonald D., Navas-Molina J.A., Kopylova E., Morton J.T., Zech Xu Z., Kightley E.P., Thompson L.R., Hyde E.R., Gonzalez A. (2017). Deblur Rapidly Resolves Single-Nucleotide Community Sequence Patterns. mSystems.

[54] Parks D.H., Tyson G.W., Hugenholtz P., Beiko R.G. (2014). STAMP: Statistical analysis of taxonomic and functional profiles. Bioinformatics.

[55] Bolger A.M., Lohse M., Usadel B. (2014). Trimmomatic: A flexible trimmer for Illumina sequence data. Bioinformatics.

[56] Dobin A., Davis C.A., Schlesinger F., Drenkow J., Zaleski C., Jha S., Batut P., Chaisson M., Gingeras T.R. (2013). STAR: Ultrafast universal RNA-seq aligner. Bioinformatics.

[57] Anders S., Pyl P.T., Huber W. (2015). HTSeq—A Python framework to work with high-throughput sequencing data. Bioinformatics.

[58] Love M.I., Huber W., Anders S. (2014). Moderated estimation of fold change and dispersion for RNA-seq data with DESeq2. Genome Biol..

[59] Young M.D., Wakefield M.J., Smyth G.K., Oshlack A. (2010). Gene ontology analysis for RNA-seq: Accounting for selection bias. Genome Biol..

[60] Mi H., Ebert D., Muruganujan A., Mills C., Albou L.-P., Mushayamaha T., Thomas P.D. (2021). PANTHER version 16: A revised family classification, tree-based classification tool, enhancer regions and extensive API. Nucleic Acids Res..

[61] Kim D.K., Lillehoj H.S., Lee S.H., Jang S.I., Bravo D. (2010). High-throughput gene expression analysis of intestinal intraepithelial lymphocytes after oral feeding of carvacrol, cinnamaldehyde, or Capsicum oleoresin. Poult. Sci..

[62] Huyghebaert G., Ducatelle R., Van Immerseel F. (2011). An update on alternatives to antimicrobial growth promoters for broilers. Veter. J..

[63] Wang J., Fan H., Han Y., Wei J., Zhao J., Zhou Z. (2016). Pyrosequencing of the broiler chicken gastrointestinal tract reveals the regional similarity and dissimilarity of microbial community. Can. J. Anim. Sci..

[64] Oakley B.B., Lillehoj H.S., Kogut M.H., Kim W.K., Maurer J.J., Pedroso A., Lee M.D., Collett S.R., Johnson T., Cox N.A. (2014). The chicken gastrointestinal microbiome. FEMS Microbiol. Lett..

[65] Choi J.H., Kim G.B., Cha C.J. (2014). Spatial heterogeneity and stability of bacterial community in the gastrointestinal tracts of broiler chickens. Poult. Sci..

[66] Gong J., Yu H., Liu T., Gill J., Chambers J., Wheatcroft R., Sabour P. (2008). Effects of zinc bacitracin, bird age and access to range on bacterial microbiota in the ileum and caeca of broiler chickens. J. Appl. Microbiol..

[67] Owens B., Tucker L., Collins M.A., McCracken K.J. (2008). Effects of different feed additives alone or in combination on broiler performance, gut microflora and ileal histology. Br. Poult. Sci..

[68] Abdelli N., Pérez J.F., Vilarrasa E., Luna I.C., Melo-Duran D., D’Angelo M., Solà-Oriol D. (2020). Targeted-Release Organic Acids and Essential Oils Improve Performance and Digestive Function in Broilers under a Necrotic Enteritis Challenge. Animals.

[69] Pham V.H., Kan L., Huang J., Geng Y., Zhen W., Guo Y., Abbas W., Wang Z. (2020). Dietary encapsulated essential oils and organic acids mixture improves gut health in broiler chickens challenged with necrotic enteritis. J. Anim. Sci. Biotechnol..

[70] Thibodeau A., Fravalo P., Yergeau E., Arsenault J., Lahaye L., Letellier A. (2015). Chicken Caecal Microbiome Modifications Induced by Campylobacter jejuni Colonization and by a Non-Antibiotic Feed Additive. PLoS ONE.

[71] Bauer B.W., Gangadoo S., Bajagai Y.S., Van T.T.H., Moore R.J., Stanley D. (2019). Oregano powder reduces Streptococcus and increases SCFA concentration in a mixed bacterial culture assay. PLoS ONE.

[72] Choi J.-H., Lee K., Kim D.-W., Kil D.Y., Kim G.-B., Cha C.-J. (2018). Influence of dietary avilamycin on ileal and cecal microbiota in broiler chickens. Poult. Sci..

[73] Yang C., Kennes Y.M., Lepp D., Yin X., Wang Q., Yu H., Yang C., Gong J., Diarra M.S. (2020). Effects of encapsulated cinnamaldehyde and citral on the performance and cecal microbiota of broilers vaccinated or not vaccinated against coccidiosis. Poult. Sci..

[74] Stanley D., Hughes R.J., Moore R.J. (2014). Microbiota of the chicken gastrointestinal tract: Influence on health, productivity and disease. Appl. Microbiol. Biotechnol..

[75] Xu S., Lin Y., Zeng D., Zhou M., Zeng Y., Wang H., Zhou Y., Zhu H., Pan K., Jing B. (2018). Bacillus licheniformis normalize the ileum microbiota of chickens infected with necrotic enteritis. Sci. Rep..

[76] Proszkowiec-Weglarz M., Miska K.B., Schreier L.L., Grim C.J., Jarvis K.G., Shao J., Vaessen S., Sygall R., Jenkins M.C., Kahl S. (2020). Research Note: Effect of butyric acid glycerol esters on ileal and cecal mucosal and luminal microbiota in chickens challenged with Eimeria maxima. Poult. Sci..

[77] Lee K.-C., Kil D.Y., Sul W.J. (2017). Cecal microbiome divergence of broiler chickens by sex and body weight. J. Microbiol..

[78] Stanley D., Hughes R.J., Geier M.S., Moore R.J. (2016). Bacteria within the Gastrointestinal Tract Microbiota Correlated with Improved Growth and Feed Conversion: Challenges Presented for the Identification of Performance Enhancing Probiotic Bacteria. Front. Microbiol..

[79] Meehan C., Beiko R.G. (2014). A Phylogenomic View of Ecological Specialization in the Lachnospiraceae, a Family of Digestive Tract-Associated Bacteria. Genome Biol. Evol..

[80] Yacoubi N., Saulnier L., Bonnin E., Devillard E., Eeckhaut V., Rhayat L., Ducatelle R., Van Immerseel F. (2018). Short-chain arabinoxylans prepared from enzymatically treated wheat grain exert prebiotic effects during the broiler starter period. Poult. Sci..

[81] Zhong H., Wang X.-G., Wang J., Chen Y.-J., Qin H.-L., Yang R. (2021). Impact of probiotics supplement on the gut microbiota in neonates with antibiotic exposure: An open-label single-center randomized parallel controlled study. World J. Pediatr..

[82] McKenna A., Ijaz U.Z., Kelly C., Linton M., Sloan W.T., Green B.D., Lavery U., Dorrell N., Wren B.W., Richmond A. (2020). Impact of industrial production system parameters on chicken microbiomes: Mechanisms to improve performance and reduce Campylobacter. Microbiome.

[83] Wang Y., Xu Y., Xu S., Yang J., Wang K., Zhan X. (2021). Bacillus subtilis DSM29784 Alleviates Negative Effects on Growth Performance in Broilers by Improving the Intestinal Health Under Necrotic Enteritis Challenge. Front. Microbiol..

[84] Yang W.Y., Lee Y., Lu H., Chou C.H., Wang C. (2018). Analysis of contributory gut microbiota and lauric acid against necrotic enteritis in Clostridium perfringens and Eimeria side-by-side challenge model. bioRxiv.

[85] Eeckhaut V., Van Immerseel F., Croubels S., De Baere S., Haesebrouck F., Ducatelle R., Louis P., Vandamme P. (2011). Butyrate production in phylogenetically diverse Firmicutes isolated from the chicken caecum. Microb. Biotechnol..

[86] Polansky O., Sekelova Z., Faldynova M., Sebkova A., Sisak F., Rychlik I. (2016). Important Metabolic Pathways and Biological Processes Expressed by Chicken Cecal Microbiota. Appl. Environ. Microbiol..

[87] Jacquier V., Nelson A., Jlali M., Rhayat L., Brinch K., Devillard E. (2019). Bacillus subtilis 29784 induces a shift in broiler gut microbiome toward butyrate-producing bacteria and improves intestinal histomorphology and animal performance. Poult. Sci..

[88] Shang Q., Liu S., He T., Liu H., Mahfuz S., Ma X., Piao X. (2020). Effects of wheat bran in comparison to antibiotics on growth performance, intestinal immunity, barrier function, and microbial composition in broiler chickens. Poult. Sci..

[89] Xue F., Shi L., Li Y., Ni A., Ma H., Sun Y., Chen J. (2020). Effects of replacing dietary Aureomycin with a combination of plant essential oils on production performance and gastrointestinal health of broilers. Poult. Sci..

[90] Ma X., Wang Q., Li H., Xu C., Cui N., Zhao X. (2017). 16S rRNA genes Illumina sequencing revealed differential cecal microbiome in specific pathogen free chickens infected with different subgroup of avian leukosis viruses. Veter. Microbiol..

[91] Chen H.-L., Zhao X.-Y., Zhao G.-X., Huang H.-B., Li H.-R., Shi C.-W., Yang W.-T., Jiang Y.-L., Wang J.-Z., Ye L.-P. (2020). Dissection of the cecal microbial community in chickens after Eimeria tenella infection. Parasites Vectors.

[92] Khan S., Chousalkar K.K. (2020). Salmonella Typhimurium infection disrupts but continuous feeding of Bacillus based probiotic restores gut microbiota in infected hens. J. Anim. Sci. Biotechnol..

[93] Hung D.-Y., Cheng Y.-H., Chen W.-J., Hua K.-F., Pietruszka A., Dybus A., Lin C.-S., Yu Y.-H. (2019). Bacillus licheniformis-Fermented Products Reduce Diarrhea Incidence and Alter the Fecal Microbiota Community in Weaning Piglets. Animals.

[94] Kulshreshtha G., Rathgeber B., MacIsaac J., Boulianne M., Brigitte L., Stratton G., Thomas N.A., Critchley A.T., Hafting J., Prithiviraj B. (2017). Feed Supplementation with Red Seaweeds, *Chondrus crispus* and *Sarcodiotheca gaudichaudii*, Reduce Salmonella Enteritidis in Laying Hens. Front. Microbiol..

[95] Aydın Ö.D., Yıldız G. (2020). Using of Essential Oil Mixture in Quail Breeders (Coturnix Coturnix Japonica) for Improving Cecal Short-Chain Fatty Acid Concentrations. Turk. J. Agric.-Food Sci. Technol..

[96] Tiihonen K., Kettunen H., Bento M., Saarinen M., Lahtinen S., Ouwehand A., Schulze H., Rautonen N. (2010). The effect of feeding essential oils on broiler performance and gut microbiota. Br. Poult. Sci..

[97] Ren H., Vahjen W., Dadi T., Saliu E.-M., Boroojeni F.G., Zentek J. (2019). Synergistic Effects of Probiotics and Phytobiotics on the Intestinal Microbiota in Young Broiler Chicken. Microorganisms.

[98] Mašek T., Starčević K. (2014). Mikulec Einfluss des zusatzes von Thymol, Gerbsäure oder Gallussäure zum Futter auf Leis-tung, Malondialdehyd-Gehalt im serum und die fermentationsleistung im Blinddarm von Broilern. Eur. Poult. Sci..

[99] Adewole D.I., Oladokun S., Santin E. (2021). Effect of organic acids–essential oils blend and oat fiber combination on broiler chicken growth performance, blood parameters, and intestinal health. Anim. Nutr..

[100] Sun B., Hou L., Yang Y. (2020). Effects of Altered Dietary Fiber on the Gut Microbiota, Short-Chain Fatty Acids and Cecum of Chickens during Different Growth Periods. Anim. Sci. Zool.-Prepr..

[101] Bajagai Y., Radovanovic A., Steel J., Stanley D. (2021). The Effects of Continual Consumption of *Origanum Vulgare* on Liver Transcriptomics. Animals.

[102] You X., Xu M., Li Q., Zhang K., Hao G., Xu H. (2019). Discovery of potential transcriptional biomarkers in broiler chicken for detection of amantadine abuse based on RNA sequencing technology. Food Addit. Contam. Part A Chem. Anal. Control. Expo. Risk Assess..

[103] Hong Y., Cheng Y., Guan L., Zhou Z., Li X., Shi D., Xiao Y. (2021). *Bacillus amyloliquefaciens* TL Downregulates the Ileal Expression of Genes Involved in Immune Responses in Broiler Chickens to Improve Growth Performance. Microorganisms.

[104] González-Mariscal L., Betanzos A., Nava P., Jaramillo B. (2003). Tight junction proteins. Prog. Biophys. Mol. Biol..

[105] Wada A.M., Reese D.E., Bader D.M. (2001). Bves: Prototype of a new class of cell adhesion molecules expressed during coronary artery development. Development.

[106] Osler M.E., Chang M.S., Bader D.M. (2005). Bves modulates epithelial integrity through an interaction at the tight junction. J. Cell Sci..

[107] Russ P.K., Kupperman A.I., Presley S.-H., Haselton F.R., Chang M.S. (2010). Inhibition of RhoA Signaling with Increased Bves in Trabecular Meshwork Cells. Investig. Opthalmol. Vis. Sci..

[108] A Hager H., Bader D.M. (2009). Bves: Ten years after. Histol. Histopathol..

[109] Vasavada T.K., DiAngelo J.R., Duncan M.K. (2004). Developmental Expression of Pop1/Bves. J. Histochem. Cytochem..

[110] Andrée B., Fleige A., Arnold H.-H., Brand T. (2002). Mouse Pop1 Is Required for Muscle Regeneration in Adult Skeletal Muscle. Mol. Cell. Biol..

[111] DiAngelo J.R., Vasavada T.K., Cain W., Duncan M.K. (2001). Production of Monoclonal Antibodies Against Chicken Pop1 (BVES). Hybrid. Hybridomics.

[112] Gu J., Liang Q., Liu C., Li S. (2020). Genomic Analyses Reveal Adaptation to Hot Arid and Harsh Environments in Native Chickens of China. Front. Genet..

[113] Froese A., Breher S.S., Waldeyer C., Schindler R.F., Nikolaev V.O., Rinné S., Wischmeyer E., Schlueter J., Becher J., Simrick S. (2012). Popeye domain containing proteins are essential for stress-mediated modulation of cardiac pacemaking in mice. J. Clin. Investig..

[114] Torlopp A., Breher S.S., Schlüter J., Brand T. (2006). Comparative analysis of mRNA and protein expression of Popdc1 (Bves) during early development in the chick embryo. Dev. Dyn..

[115] Aengwanich W., Simaraks S. (2004). Pathology of heart, lung, liver and kidney in broilers under chronic heat stress. Songklanakarin J. Sci. Technol..

[116] Glahn R.P., Mitsos W.J., Wideman R.F. (1987). Evaluation of Sex Differences in Embryonic Heart Rates. Poult. Sci..

[117] Ringer R.K., Weiss H.S., Sturkie P.D. (1957). Heart Rate of Chickens as Influenced by Age and Gonadal Hormones. Am. J. Physiol. Content.

[118] Baillat D., Hakimi M.-A., Näär A.M., Shilatifard A., Cooch N., Shiekhattar R. (2005). Integrator, a Multiprotein Mediator of Small Nuclear RNA Processing, Associates with the C-Terminal Repeat of RNA Polymerase II. Cell.

[119] Will C.L., Lührmann R. (2011). Spliceosome structure and function. Cold Spring Harb. Perspect. Biol..

[120] Tsalikis J., Tattoli I., Ling A., Sorbara M.T., Croitoru D.O., Philpott D.J., Girardin S.E. (2015). Intracellular Bacterial Pathogens Trigger the Formation of U Small Nuclear RNA Bodies (U Bodies) through Metabolic Stress Induction. J. Biol. Chem..

[121] Chittka D., Banas B., Lennartz L., Putz F.J., Eidenschink K., Beck S., Stempfl T., Moehle C., Reichelt-Wurm S., Banas M.C. (2018). Long-term expression of glomerular genes in diabetic nephropathy. Nephrol. Dial. Transplant..

[122] Wright E.M. (2013). Glucose transport families SLC5 and SLC50. Mol. Asp. Med..

[123] Leturque A., Brot-Laroche E., Le Gall M., Stolarczyk E., Tobin V. (2005). The role of GLUT2 in dietary sugar handling. J. Physiol. Biochem..

[124] Fukuzawa T., Fukazawa M., Ueda O., Shimada H., Kito A., Kakefuda M., Kawase Y., Wada N.A., Goto C., Fukushima N. (2013). SGLT5 Reabsorbs Fructose in the Kidney but Its Deficiency Paradoxically Exacerbates Hepatic Steatosis Induced by Fructose. PLoS ONE.

[125] Gaby A.R. (2005). Adverse effects of dietary fructose. Altern. Med. Rev. J. Clin. Ther..

[126] Boles M.K., Wilkinson B.M., Wilming L.G., Liu B., Probst F.J., Harrow J., Grafham D., Hentges K.E., Woodward L.P., Maxwell A. (2009). Discovery of Candidate Disease Genes in ENU-Induced Mouse Mutants by Large-Scale Sequencing, Including a Splice-Site Mutation in Nucleoredoxin. PLoS Genet..

[127] Ito M., Yuan C.-X., Okano H.J., Darnell R., Roeder R.G. (2000). Involvement of the TRAP220 Component of the TRAP/SMCC Coactivator Complex in Embryonic Development and Thyroid Hormone Action. Mol. Cell.

[128] Ito M., Okano H.J., Darnell R.B., Roeder R.G. (2002). The TRAP100 component of the TRAP/Mediator complex is essential in broad transcriptional events and development. EMBO J..

[129] Wolton K., Barnes E., Wright J., Hentges K. (2014). Two alleles of Med31 provide a model to study delayed fetal growth and endochondral ossification. Toxicol. Lett..

[130] Kim S.K., Brotslaw E., Thome V., Mitchell J., Ventrella R., Collins C., Mitchell B. (2020). A role for Cep70 in centriole amplification in multiciliated cells. Dev. Biol..

[131] Yang Y., Ran J., Liu M., Li D., Li Y., Shi X., Meng D., Pan J., Ou G., Aneja R. (2014). CYLD mediates ciliogenesis in multiple organs by deubiquitinating Cep70 and inactivating HDAC6. Cell Res..

[132] Shi X., Yao Y., Wang Y., Zhang Y., Huang Q., Zhou J., Liu M., Li D. (2015). Cep70 regulates microtubule stability by interacting with HDAC6. FEBS Lett..

[133] Shi X., Wang J., Yang Y., Ren Y., Zhou J., Li D. (2012). Cep70 promotes microtubule assembly in vitro by increasing microtubule elongation. Acta Biochim. Biophys. Sin..

[134] Nigg E.A., Raff J. (2009). Centrioles, Centrosomes, and Cilia in Health and Disease. Cell.

[135] Libe R., Horvath A., Vezzosi D., Fratticci A., Coste J., Perlemoine K., Ragazzon B., Guillaud-Bataille M., Groussin L., Clauser E. (2011). Frequent Phosphodiesterase 11A Gene (PDE11A) Defects in Patients with Carney Complex (CNC) Caused byPRKAR1AMutations:PDE11AMay Contribute to Adrenal and Testicular Tumors in CNC as a Modifier of the Phenotype. J. Clin. Endocrinol. Metab..

[136] Faucz F.R., Horvath A., Rothenbuhler A., Almeida M.Q., Libe R., Raffin-Sanson M.-L., Bertherat J., Carraro D., Soares F.A., Molina G.D.C. (2011). Phosphodiesterase 11A (PDE11A) Genetic Variants May Increase Susceptibility to Prostatic Cancer. J. Clin. Endocrinol. Metab..

[137] Dietert K., Reppe K., Mundhenk L., Witzenrath M., Gruber A.D. (2014). mCLCA3 Modulates IL-17 and CXCL-1 Induction and Leukocyte Recruitment in Murine Staphylococcus aureus Pneumonia. PLoS ONE.

[138] Mamber S.W., Gurel V., Lins J., Ferri F., Beseme S., McMichael J. (2020). Effects of cannabis oil extract on immune response gene expression in human small airway epithelial cells (HSAEpC): Implications for chronic obstructive pulmonary disease (COPD). J. Cannabis Res..

[139] Farmahin R., Gannon A.M., Gagné R., Rowan-Carroll A., Kuo B., Williams A., Curran I., Yauk C.L. (2019). Hepatic transcriptional dose-response analysis of male and female Fischer rats exposed to hexabromocyclododecane. Food Chem. Toxicol..

[140] Kwon T.-J., Kim D.-B., Bae J.W., Sagong B., Choi S.-Y., Cho H.-J., Kim U.-K., Lee K.-Y. (2014). Molecular cloning, characterization, and expression of pannexin genes in chicken. Poult. Sci..

[141] Tang W., Ahmad S., Shestopalov V.I., Lin X. (2008). Pannexins are new molecular candidates for assembling gap junctions in the cochlea. NeuroReport.

[142] Jiang J.X., Penuela S. (2016). Connexin and pannexin channels in cancer. BMC Cell Biol..

[143] Xie C.-R., Sun H., Wang F.-Q., Li Z., Yin Y.-R., Fang Q.-L., Sun Y., Zhao W.-X., Zhang S., Wang X.-M. (2015). Integrated analysis of gene expression and DNA methylation changes induced by hepatocyte growth factor in human hepatocytes. Mol. Med. Rep..

[144] Anand P., Kumar S.V., Ravi K., Simmi T. (2021). Differential gene expression in duodenum of colored broiler chicken divergently selected for residual feed intake. Trop. Anim. Health Prod..

[145] Ren J., Li Y., Xu N., Li H., Li C., Han R., Wang Y., Li Z., Kang X., Liu X. (2017). Association of estradiol on expression of melanocortin receptors and their accessory proteins in the liver of chicken (*Gallus gallus*). Gen. Comp. Endocrinol..

[146] Blankenship K., Gilley A., Piekarski A., Orlowski S., Greene E., Bottje W., Anthony N., Dridi S. (2016). Differential expression of feeding-related hypothalamic neuropeptides in the first generation of quails divergently selected for low or high feed efficiency. Neuropeptides.

[147] Wilson C., Lin J.E., Li P., Snook A., Gong J., Sato T., Liu C., Girondo M.A., Rui H., Hyslop T. (2014). The Paracrine Hormone for the GUCY2C Tumor Suppressor, Guanylin, Is Universally Lost in Colorectal Cancer. Cancer Epidemiol. Biomark. Prev..

[148] Steinbrecher K.A., Harmel-Laws E., Garin-Laflam M.P., Mann E.A., Bezerra L.D., Hogan S.P., Cohen M.B. (2011). Murine Guanylate Cyclase C Regulates Colonic Injury and Inflammation. J. Immunol..

[149] Santos A., Giráldez F.J., Trevisi E., Lucini L., Frutos J., Andrés S. (2018). Liver transcriptomic and plasma metabolomic profiles of fattening lambs are modified by feed restriction during the suckling period1. J. Anim. Sci..

[150] Erwin E.A., Jaramillo L.M., Smith B., Kruszewski P.G., Kahwash B., Grayson M.H., Mejias A., Ramilo O. (2021). Sex Differences in Blood Transcriptional Profiles and Clinical Phenotypes in Pediatric Patients with Eosinophilic Esophagitis. J. Allergy Clin. Immunol. Pract..

[151] Shaik A.P., AlSaeed A.H., Kiranmayee S., Bammidi V., Sultana A. (2013). Phylogenetic analysis of cubilin (CUBN) gene. Bioinformation.

[152] Christensen E.I., Birn H. (2002). Megalin and cubilin: Multifunctional endocytic receptors. Nat. Rev. Mol. Cell Biol..

[153] Lee J., Karnuah A.B., Rekaya R., Anthony N.B., Aggrey S.E. (2015). Transcriptomic analysis to elucidate the molecular mechanisms that underlie feed efficiency in meat-type chickens. Mol. Gen. Genet. MGG.

[154] Sun H., Jiang R., Xu S., Zhang Z., Xu G., Zheng J., Qu L. (2015). Transcriptome responses to heat stress in hypothalamus of a meat-type chicken. J. Anim. Sci. Biotechnol..

[155] Balla T. (2013). Phosphoinositides: Tiny Lipids with Giant Impact on Cell Regulation. Physiol. Rev..

[156] Srivastava S., Li Z., Lin L., Liu G., Ko K., Coetzee W.A., Skolnik E.Y. (2005). The Phosphatidylinositol 3-Phosphate Phosphatase Myotubularin- Related Protein 6 (MTMR6) Is a Negative Regulator of the Ca^2+^ -Activated K^+^ Channel K Ca 3.1. Mol. Cell. Biol..

[157] Cheng Y., Gu X., Bednarczyk P., Wiedemann F., Haddad G., Siemen D. (2008). Hypoxia Increases Activity of the BK-Channel in the Inner Mitochondrial Membrane and Reduces Activity of the Permeability Transition Pore. Cell. Physiol. Biochem..

[158] Li L., Lu D.-Z., Li Y.-M., Zhang X.-Q., Zhou X.-X., Jin X. (2014). Proteomic analysis of liver mitochondria from rats with nonalcoholic steatohepatitis. World J. Gastroenterol..

[159] Huang Z., Jin S., Lv Z. (2021). Dietary genistein supplementation alters mRNA expression profile and alternative splicing signature in the thymus of chicks with lipopolysaccharide challenge. Poult. Sci..

[160] Smith I.A., Knezevic B.R., Ammann J.U., Rhodes D.A., Aw D., Palmer D.B., Mather I.H., Trowsdale J. (2010). BTN1A1, the Mammary Gland Butyrophilin, and BTN2A2 Are Both Inhibitors of T Cell Activation. J. Immunol..

[161] Yamazaki T., Goya I., Graf D., Craig S., Martin-Orozco N., Dong C. (2010). A Butyrophilin Family Member Critically Inhibits T Cell Activation. J. Immunol..

[162] Wu C., Liu L., Jinglong H., Lianjun L., Yongwang M. (2014). Isolation, bioinformatic and tissue expression analysis of a novel water buffalo gene-BTN1A1. Buffalo Bull..

[163] Xu Z., Le K., Moghadasian M.H. (2008). Long-term phytosterol treatment alters gene expression in the liver of apo E-deficient mice. J. Nutr. Biochem..

[164] Moghadasian M.H. (2000). Pharmacological properties of plant sterols: In vivo and in vitro observations. Life Sci..

[165] Wolfs M., De Jong N., Ocké M.C., Verhagen H., Verschuren W.M. (2006). Effectiveness of customary use of phytosterol/-stanol enriched margarines on blood cholesterol lowering. Food Chem. Toxicol..

[166] Zhang Y., Yang Y., Zhang Y., Liu Z. (2021). lncRNA ST8SIA6-AS1 facilitates proliferation and invasion in liver cancer by regulating miR-142-3p. Exp. Ther. Med..

[167] Bonnet L., Karkeni E., Couturier C., Astier J., Dalifard J., Defoort C., Svilar L., Martin J.-C., Tourniaire F., Landrier J.-F. (2018). Gene Expression Pattern in Response to Cholecalciferol Supplementation Highlights Cubilin as a Major Protein of 25(OH)D Uptake in Adipocytes and Male Mice White Adipose Tissue. Endocrinology.

[168] Nykjaer A., Fyfe J.C., Kozyraki R., Leheste J.-R., Jacobsen C., Nielsen M.S., Verroust P.J., Aminoff M., de la Chapelle A., Moestrup S.K. (2001). Cubilin dysfunction causes abnormal metabolism of the steroid hormone 25(OH) vitamin D3. Proc. Natl. Acad. Sci. USA.

[169] Collison K.S., Maqbool Z., Saleh S.M., Inglis A., Makhoul N.J., Bakheet R., Al-Johi M., Al-Rabiah R., Zaidi M.Z., Al-Mohanna F.A. (2009). Effect of dietary monosodium glutamate on trans fat-induced nonalcoholic fatty liver disease. J. Lipid Res..

