# Supplementary material for: Microbiota and Transcriptomic Effects of an Essential Oil Blend and Its Delivery Route Compared to an Antibiotic Growth Promoter in Broiler Chickens
Source: Microorganisms. 2022 Apr 21;10(5):861. doi: 10.3390/microorganisms10050861 (PMC9147064; doi:10.3390/microorganisms10050861)
Supplement: Supplementary file 1 [file microorganisms-10-00861-s001.zip › microorganisms-1644589-supplementary.pdf]

Supplementary Material

Table S1. Ingredients, calculated, and analyzed compositions of experimental diets[1] (as-fed basis, percentage (%), unless otherwise stated).

Ingredients	Phases			
	Starter (0–14 d)		Grower (15–28 d)	
	Control diet	Antibiotic diet	Control diet	Antibiotic diet
	Ingredient Composition			
Corn (ground)	51.08	50.98	45.36	45.25
Soybean meal-46.5	41.44	41.45	36.31	36.33
Wheat	-	-	10	10
Animal/vegetable fat	2.93	2.97	4.22	4.26
Limestone	1.80	1.80	1.65	1.65
Dicalcium Phosphate	1.24	1.24	1.06	1.06
DL Methionine premix[2]	0.59	0.59	0.53	0.53
Vitamin/Mineral Premix [3, 4]	0.50	0.50	0.50	0.50
Salt	0.41	0.41	0.37	0.37
Lysine HCl	0.01	0.01	0.00	0.00
BMD 110G[5]	-	0.05	-	0.05
Total	100	100	100	100
Nutrient	Calculated composition			
Metabolizable energy (kcal/kg)	3,000	3,000	3,100	3,100
Crude protein	23	23	21.5	21.5
Calcium	0.96	0.96	0.87	0.87
Available phosphorus	0.48	0.48	0.44	0.44
Sodium	0.19	0.19	0.18	0.18
Digestible lysine	1.28	1.28	1.15	1.16
Digestible methionine + cysteine	0.95	0.95	0.87	0.87
Digestible Tryptophan	0.25	0.25	0.23	0.23
Digestible Threonine	0.89	0.89	0.82	0.82
	Analyzed composition			
Dry Matter	90.7	90.8	93.2	93.5
Crude protein	24.8	25	22.5	23.8
Crude fat	5.50	5.79	6.84	6.85
Calcium	1.06	1.13	1.00	0.96
Potassium	1.14	1.16	0.99	1.04
Phosphorus	0.69	0.70	0.67	0.62
Sodium	0.19	0.21	0.21	0.16

[1] Basal diet (NC); antibiotic diet containing NC + 0.05% bacitracin methylene disalicylate (BMD). [2] Supplied/kg premix: DL-Methionine, 0.5 kg; wheat middling, 0.5 kg. [3] Starter vitamin-mineral premix contained the following per kg of diet: 9750 IU vitamin A; 2000 IU vitamin D3; 25 IU vitamin E; 2.97 mg vitamin K; 7.6 mg riboflavin; 13.5 mg Dl Ca-pantothenate; 0.012 mg vitamin B12; 29.7 mg niacin; 1.0 mg folic acid, 801 mg choline; 0.3 mg biotin; 4.9 mg pyridoxine; 2.9 mg thiamine; 70.2 mg manganese; 80.0 mg zinc; 25 mg copper; 0.15 mg selenium; 50 mg ethoxyquin; 1543 mg wheat middling's; 500 mg ground limestone. [4] Grower and Finisher vitamin-mineral premix contained the following per kg of diet: 9750 IU vitamin A; 2000 IU vitamin D3; 25 IU vitamin E; 2.97 mg vitamin K; 7.6 mg riboflavin; 13.5 mg Dl Ca-pantothenate; 0.012 mg vitamin B12; 29.7 mg niacin; 1.0 mg folic acid, 801 mg choline; 0.3 mg biotin; 4.9 mg pyridoxine; 2.9 mg thiamine; 70.2 mg manganese; 80.0 mg zinc; 25 mg copper; 0.15 mg selenium; 50 mg ethoxyquin; 1543 mg wheat middling's; 500 mg ground limestone. [5] Bacitracin methylene disalicylate (providing 55 mg/kg mixed feed); Alpharma, Inc., Fort Lee, NJ, USA.

Table S2. Sequencing data quality control metrics for 48 liver samples for 6 treatment groups[1].

Sample	Raw Reads #	Surviving Reads #	Surviving %	Mapped Reads #	Mapped %
10D	128931886	128864410	99.95	123496072	95.83
11F	123815424	123749336	99.95	117546419	94.99
12E	100217620	100171610	99.95	95675887	95.51
13A	91263880	91218332	99.95	87621362	96.06
14E	102874686	102803518	99.93	98365003	95.68
15F	89610956	89558286	99.94	85844381	95.85
16B	103696522	103649450	99.95	99566563	96.06
17D	102727142	102673518	99.95	97718554	95.17
18C	102658094	102589816	99.93	97517267	95.06
19E	114613450	114541804	99.94	109055322	95.21
1B	111989480	111935440	99.95	107217188	95.78
20B	105128716	105083912	99.96	101140738	96.25
21D	86558618	86518732	99.95	82682287	95.57
22A	86201504	86143930	99.93	82400996	95.66
23F	97189216	97135594	99.94	92538383	95.27
25F	129747010	129692014	99.96	124312086	95.85
26D	98609268	98564006	99.95	94445790	95.82
27A	103167822	103122160	99.96	98995073	96.00
28E	119433662	119384804	99.96	113827077	95.34
29C	71048832	71015376	99.95	67880932	95.59
2E	122294110	122221882	99.94	116442856	95.27
30B	100435658	100385796	99.95	96299990	95.93
31D	110590420	110541296	99.96	106048032	95.94
32C	157571810	157485346	99.95	150219923	95.39
33A	103717506	103676040	99.96	99480220	95.95
34E	114963742	114911374	99.95	109633108	95.41
35F	99621346	99571764	99.95	94015694	94.42
36B	89037488	88999280	99.96	85651443	96.24
37E	93691904	93644694	99.95	89307326	95.37
38A	74565050	74529310	99.95	71237815	95.58
39F	96777946	96716112	99.94	92174462	95.30
3D	115361710	115292412	99.94	110464518	95.81
40D	81332670	81290328	99.95	77473448	95.30
41B	78153658	78115760	99.95	74718506	95.65
42C	89136784	89093766	99.95	85224056	95.66
43B	84600692	84556594	99.95	80853617	95.62
44E	106835414	106789686	99.96	102394089	95.88
45A	95541636	95493314	99.95	91460060	95.78
46F	76636156	76598132	99.95	73012433	95.32
47C	112641994	112573638	99.94	107374095	95.38
48D	94773314	94702008	99.92	90015678	95.05
4A	93142656	93092202	99.95	88266669	94.82
5C	102491666	102433822	99.94	97796357	95.47
6F	98781198	98720274	99.94	94109603	95.33
7B	834450506	833996014	99.95	798312414	95.72
8A	851554080	851180034	99.96	812162674	95.42
9C	123838248	123780280	99.95	118300543	95.57
24C	88404200	88369454	99.96	84734829	95.89

[1] Treatments include- A) Negative Control treatment- chicks fed a basal corn-soybean meal-wheat–based diet; B) In-feed antibiotics- chicks fed NC + 0.05% bacitracin methylene disalicylate and C) In-water essential oil- chicks supplied the essential oil via the water route at the recommended dosage of 250 mL/1000 L of drinking water; (D) In ovo saline treatment- eggs injected with 0.2 mL of physiological saline (0.9% NaCl); (E) *In ovo* essential oil treatment- eggs injected with 0.2mL of a saline + essential oil blend mixture at a dilution ratio of 2:1, F) *In ovo* + in-water essential oil treatment- chicks offered the essential oil blend via the *in ovo* and in water route, successively.

Table S3. Differentially expressed genes in the liver of broiler chickens as influenced by sex and treatment groups[1].

Sex / Treatments	Gene Symbol	Gene Description	Expression level	log2Fold-Change	*P* Value	GO Terms	KEGG Pathways
**Males (n=28)**							
B vs. A	INTS2	integrator complex subunit 2	Up	0.5	<0.01	snRNA 3'-end processing,	Genetic information processing (Spliceosome)
	SLC5A10	solute carrier family 5-member 10	Up	0.7	0.01	glucose transmembrane transport	Signaling and cellular processes (Sodium glucose cotransporter)
	MED13	mediator complex subunit 13	Up	0.6	0.01	mediator complex	Transcription machinery (RNA polymerase II system
	CEP70	centrosomal protein 70	Up	0.4	0.01	gamma-tubulin binding	Chromosome and associated proteins
	BVES	blood vessel epicardial substance	Down	-0.5	0.01	regulation of microtubule cytoskeleton organization	Tight junction
	PDE11A	phosphodiesterase 11A	Down	-0.5	0.02	3',5'-cyclic-nucleotide phosphodiesterase activity	Phosphoric-diester hydrolases
	CLCA1	chloride channel accessory 1	Down	-0.5	<0.01	intracellular calcium activated chloride channel activity	Signaling and cellular processes (Ion channels)
C vs. A	BVES	blood vessel epicardial substance	Down	-0.6	0.01	regulation of microtubule cytoskeleton organization	Tight junction
E vs. A	BVES	blood vessel epicardial substance	Down	-0.6	<0.01	regulation of microtubule cytoskeleton organization	Tight junction
**Females (n=20)**							
B vs. A	PANX2	pannexin 2	Up	1.1	<0.01	plasma membrane	signaling and cellular processes (Pores ion channels)
C vs. A	GUCY2C	guanylate cyclase 2C	Up	1.1	0.02	peptide hormone binding	Purine metabolism
	MC5R	melanocortin 5 receptor	Down	-0.9	0.02	melanocortin receptor activity	Neuroactive ligand-receptor interaction (Signalling molecules and interaction)
F vs. A	MTMR6	myotubularin related protein 6	Down	-0.6	0.02	peptidyl-tyrosine dephosphorylation	Phosphatidylinositol signaling system (Inositol phosphate metabolism)

[1] Treatments include- A) Negative Control treatment- chicks fed a basal corn-soybean meal-wheat–based diet; B) In-feed antibiotics- chicks fed NC + 0.05% bacitracin methylene disalicylate and C) In-water essential oil- chicks supplied the essential oil via the water route at the recommended dosage of 250 mL/1000 L of drinking water; D) *In ovo* saline treatment- eggs injected with 0.2 mL of physiological saline (0.9% NaCl); E) *In ovo* essential oil treatment- eggs injected with 0.2mL of a saline + essential oil blend mixture at a dilution ratio of 2:1, F) *In ovo* + in-water essential oil treatment- chicks offered the essential oil blend via the *in ovo* and in water route, successively. Each comparison is specified in the format "B vs. A", where group B is compared to group A, with group A being the denominator for the comparison. Liver tissues (50–100 mg) was sampled from 8 replicate birds/treatment (independent of sex) using 1 mL TRIzol™ (Qiagen, Hilden, Germany).

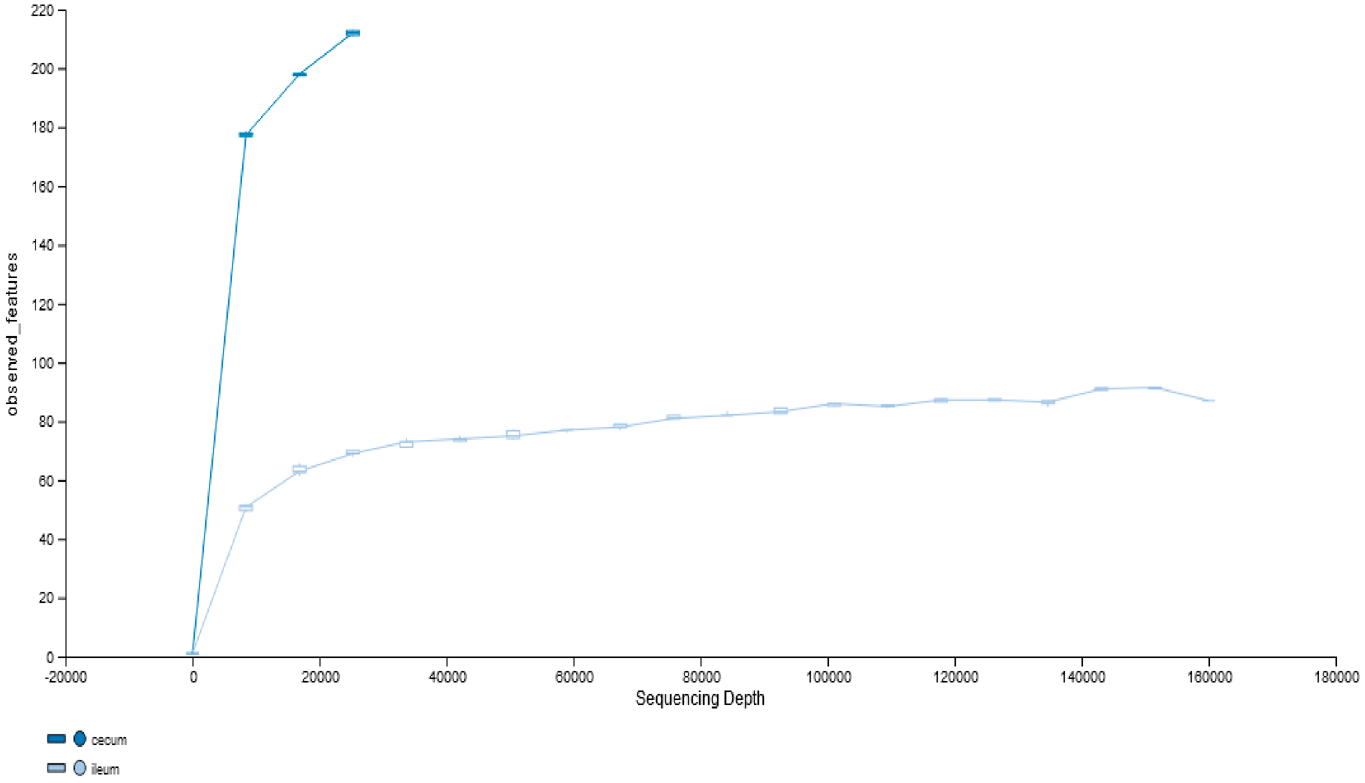

220
200
180
160
140
120
100
80
60
40
20
0
observed_features
-20000
0
20000
40000
60000
80000
100000
120000
140000
160000
180000
Sequencing Depth
cecum
ileum

(a)

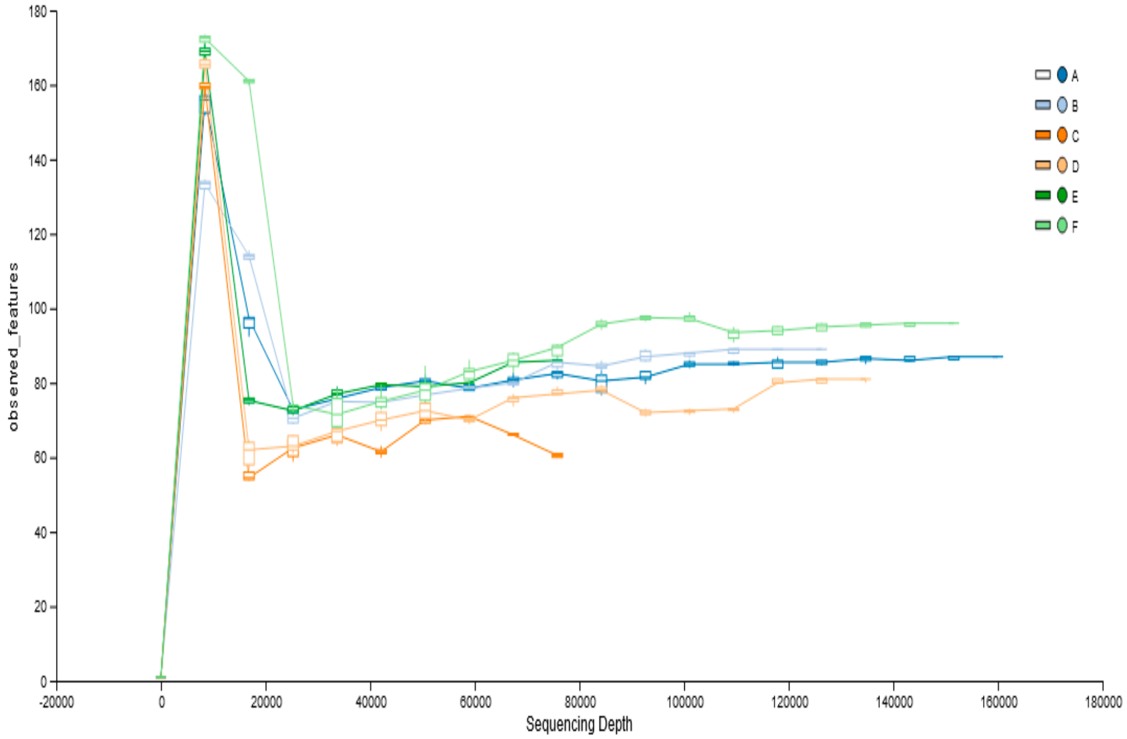

(b)

Figure S1. Rarefaction curves of observed features obtained from 16S rRNA gene V4–V5 sequences on the basis of (a)microbiota source- cecum and ileum and (b) treatment- A) chicks fed a basal corn-soybean meal-wheat–based diet (Negative Control treatment; NC); B) chicks fed NC + 0.05% bacitracin methylene disalicylate (in-feed antibiotics); C) chicks supplied the same commercial blend of EOs via the water route (in-water essential oil) at the recommended dosage of 250 mL/1000 L of drinking water; D) *in ovo* saline treatment; E) *in ovo* essential oil treatment; and F) chicks supplied EO via the *in ovo* and water route (*in ovo* + in-water essential oil treatment

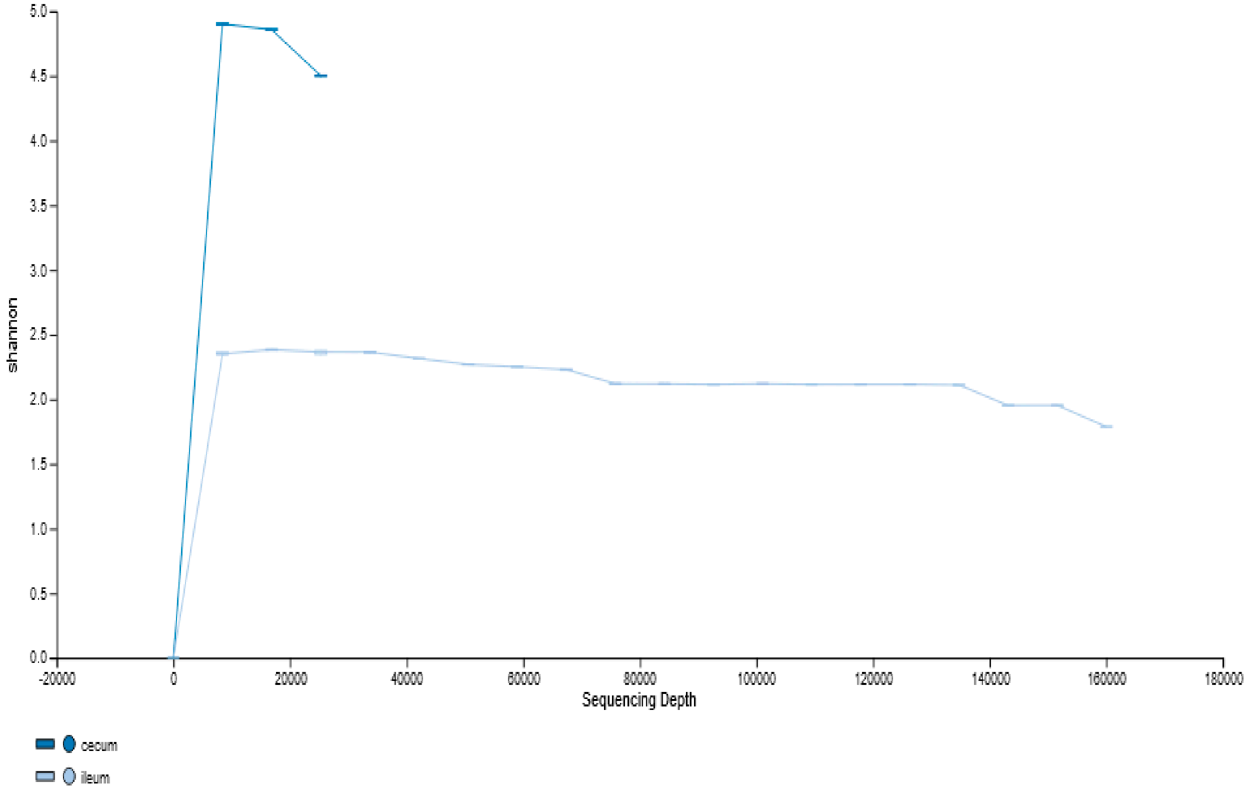

(a)

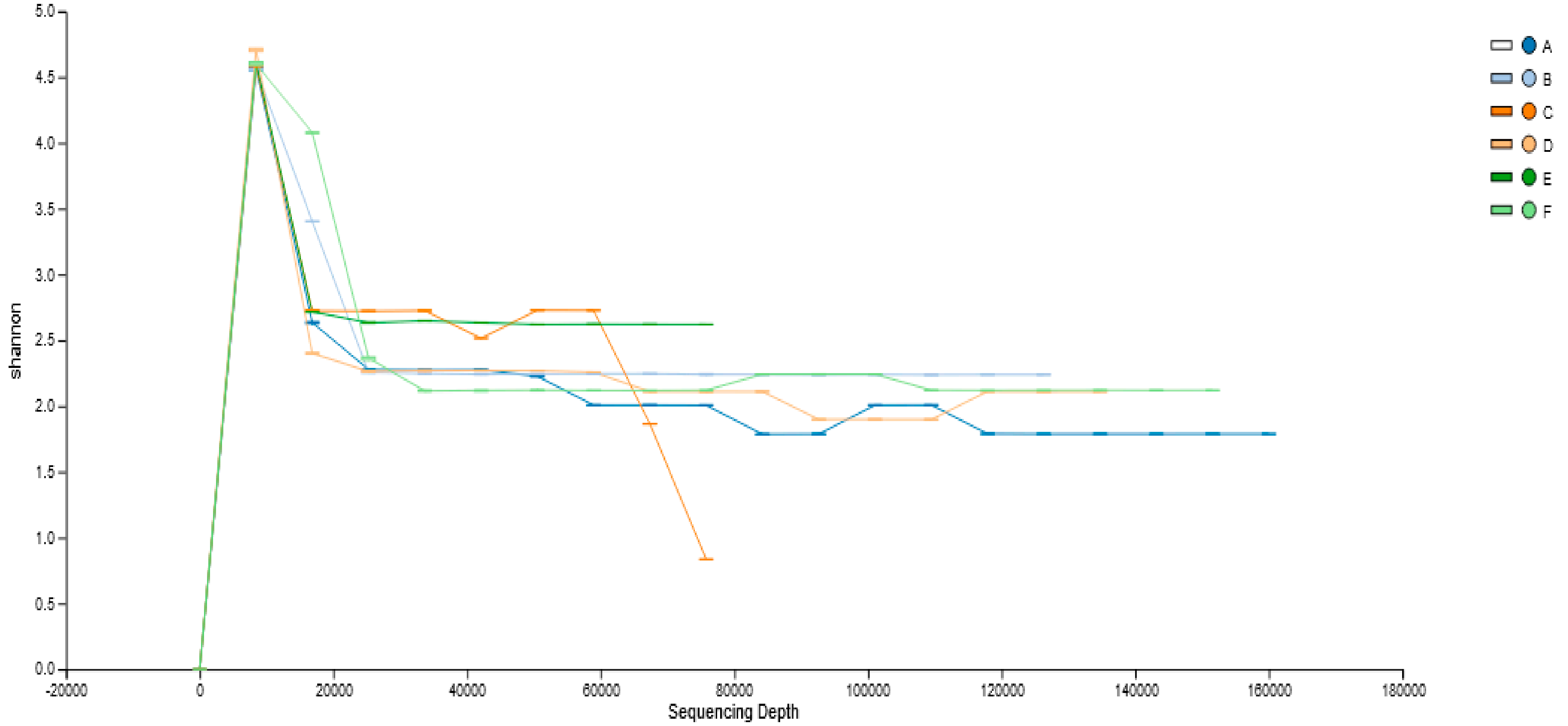

(**b**)

Figure S2. Rarefaction curves of Shannon's index obtained from 16S rRNA gene V4–V5 sequences on the basis of (a) Microbiota source- cecum and ileum and (b) Treatments, which include- A) Negative Control treatment- chicks fed a basal corn-soybean meal-wheat–based diet; B) In-feed antibiotics- chicks fed NC + 0.05% bacitracin methylene disalicylate and C) In-water essential oil- chicks supplied the essential oil via the water route at the recommended dosage of 250 mL/1000 L of drinking water; D) *In ovo* saline treatment- eggs injected with 0.2 mL of physiological saline (0.9% NaCl); E) *In ovo* essential oil treatment- eggs injected with 0.2mL of a saline + essential oil blend mixture at a dilution ratio of 2:1, F) *In ovo* + in-water essential oil treatment- chicks offered the essential oil blend via the *in ovo* and in water route, successively.

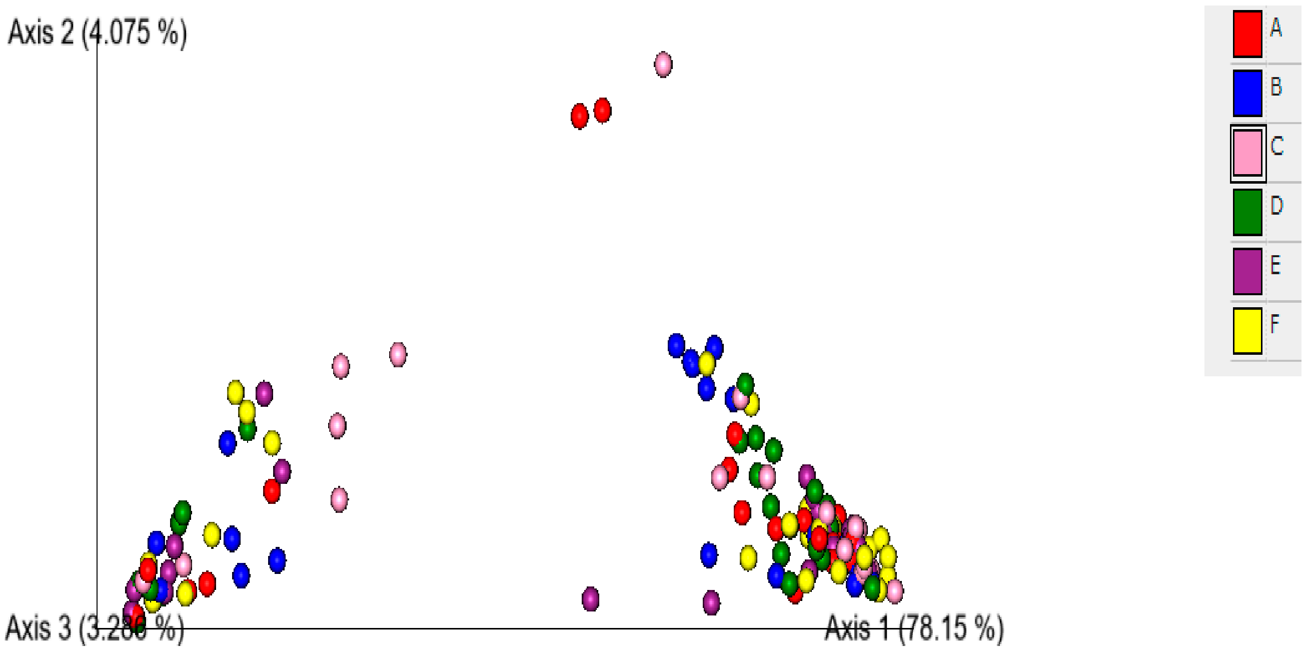

Figure S3. PCoA plots based on unweighted UniFrac metric. Each color represents a different comparison of interest. Ileum samples clustered to the left and ceca samples clustered to the right. Treatments include- A) Red: Negative Control treatment- chicks fed a basal corn-soybean meal-wheat–based diet; B) Blue: In-feed antibiotics- chicks fed NC + 0.05% bacitracin methylene disalicylate and C) Pink: In-water essential oil- chicks supplied the essential oil via the water route at the recommended dosage of 250 mL/1000 L of drinking water; D)Green: *In ovo* saline treatment- eggs injected with 0.2 mL of physiological saline (0.9% NaCl); E) Purple: *In ovo* essential oil treatment- eggs injected with 0.2mL of a saline + essential oil blend mixture at a dilution ratio of 2:1, F) Yellow: *In ovo* + in-water essential oil treatment- chicks offered the essential oil blend via the *in ovo* and in water route, successively.

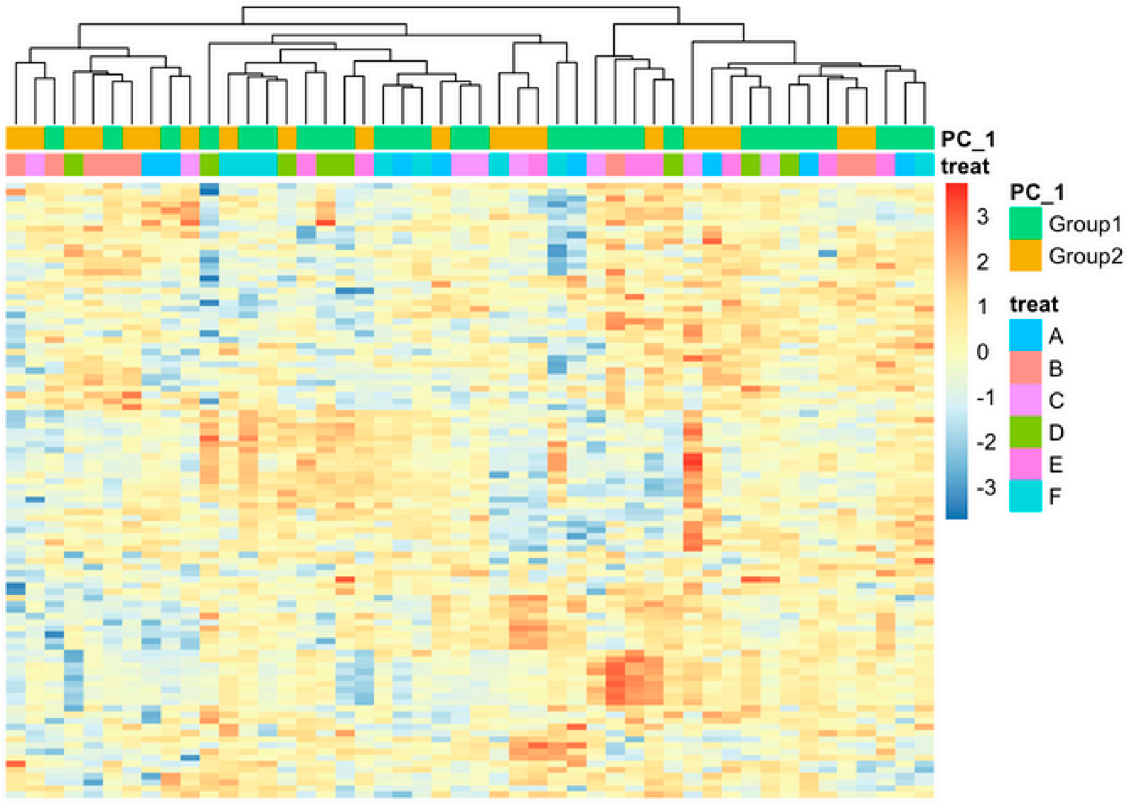

Figure S4. Heatmaps of the top 100 most variable genes. Orange = low expression, blue = high expression Treatments include- A) Negative Control treatment- chicks fed a basal corn-soybean meal-wheat–based diet; B) In-feed antibiotics- chicks fed NC + 0.05% bacitracin methylene disalicylate and C) In-water essential oil- chicks supplied the essential oil via the water route at the recommended dosage of 250 mL/1000 L of drinking water; D) *In ovo* saline treatment- eggs injected with 0.2 mL of physiological saline (0.9% NaCl); E) *In ovo* essential oil treatment- eggs injected with 0.2mL of a saline + essential oil blend mixture at a dilution ratio of 2:1, F) *In ovo* + in-water essential oil treatment- chicks offered the essential oil blend via the *in ovo* and in water route, successively.

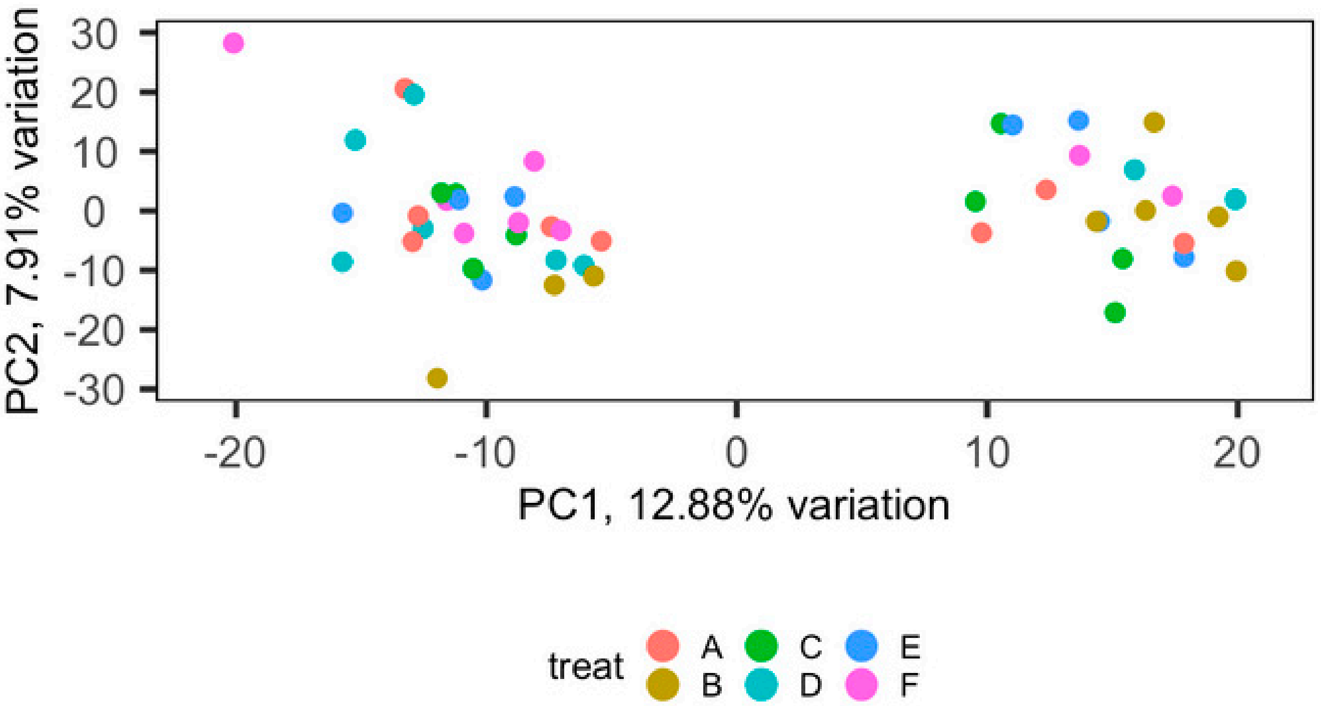

Figure S5. Principal component analysis (PCA) was performed with R package gmodels. The more similar the treatment, the closer the distance reflected in PCA. Treatments include- A) Negative Control treatment- chicks fed a basal corn-soybean meal-wheat–based diet; B) In-feed antibiotics- chicks fed NC + 0.05% bacitracin methylene disalicylate and C) In-water essential oil- chicks supplied the essential oil via the water route at the recommended dosage of 250 mL/1000 L of drinking water; D) *In ovo* saline treatment- eggs injected with 0.2 mL of physiological saline (0.9% NaCl); E) *In ovo* essential oil treatment- eggs injected with 0.2mL of a saline + essential oil blend mixture at a dilution ratio of 2:1, F) *In ovo* + in-water essential oil treatment- chicks offered the essential oil blend via the *in ovo* and in water route, successively.

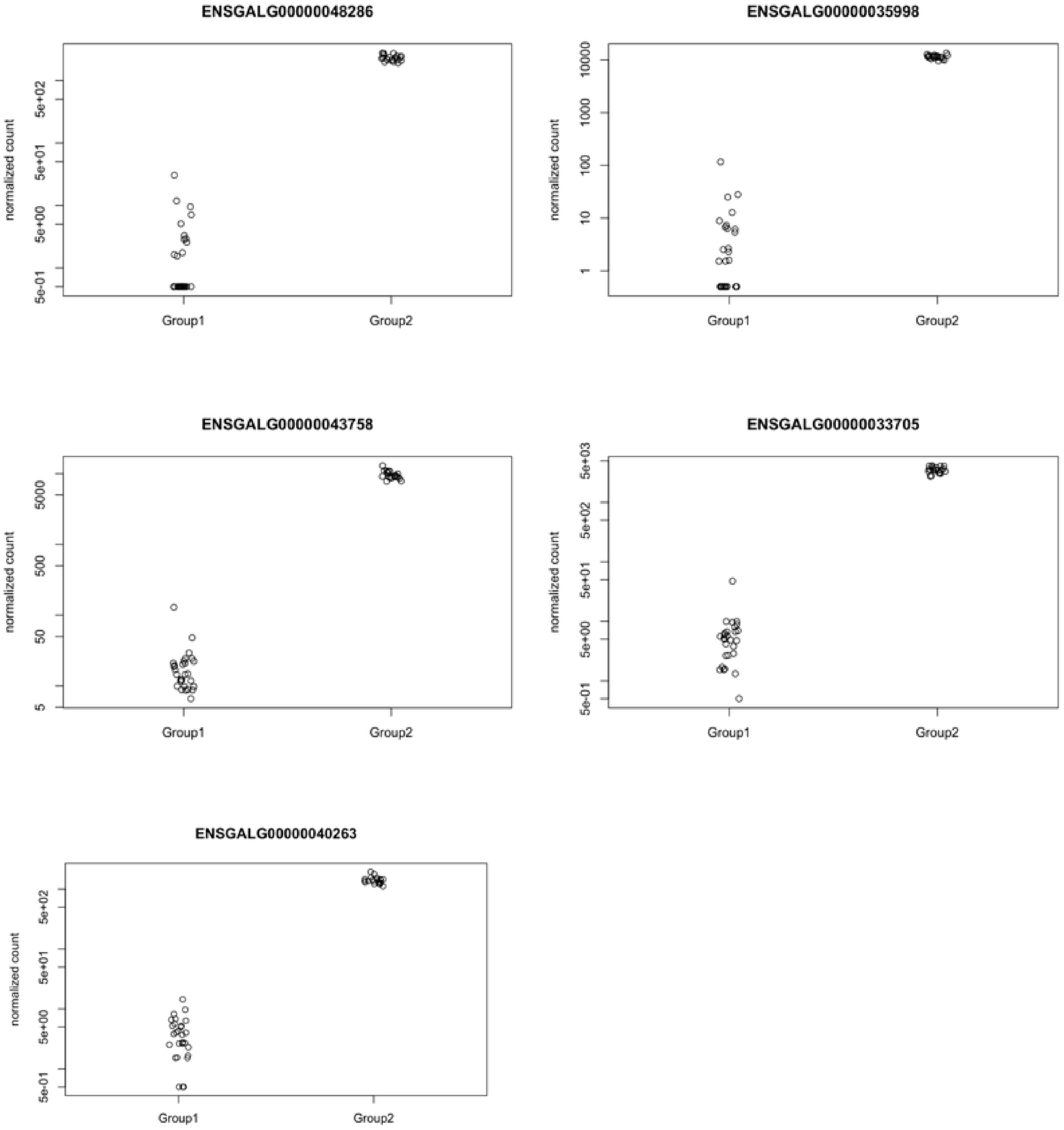

Figure S6. Gene expression of chrW gene shows much higher expression in Group2 than Group1, indicating that Group2 samples are probably females (n=20) and Group1 samples are probably males (n=28). Group2 are samples with PC1 score > 0, Group 1 are samples with PC1 scores < 0.

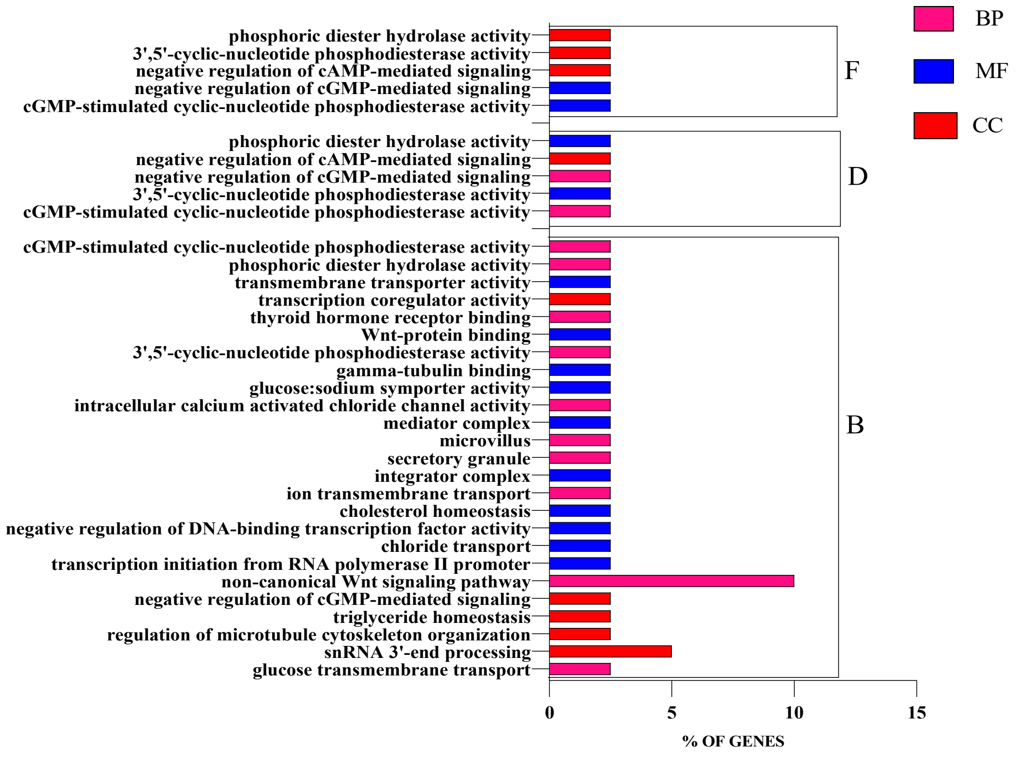

(a)

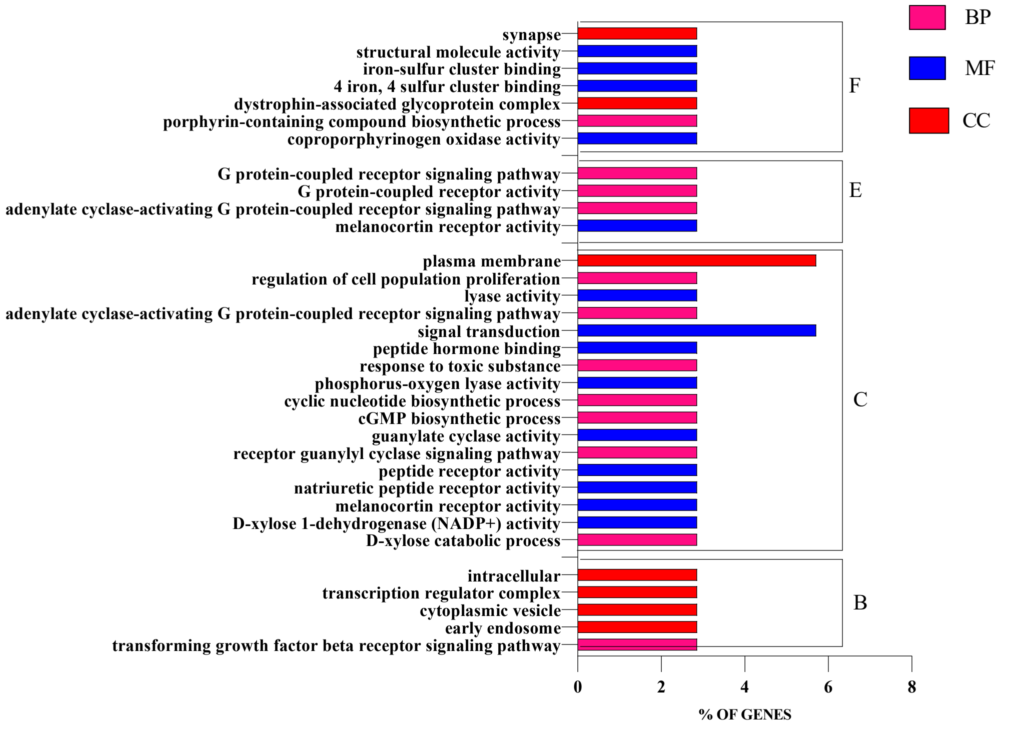

(b)

Figure S7. Gene ontology (GO) classifications of differentially expressed genes (DEGs) between treatment groups in (a) male and (b) female transcripts, respectively. Treatments groups include- A) Negative Control treatment- chicks fed a basal corn-soybean meal-wheat–based diet; B) In-feed antibiotics- chicks fed NC + 0.05% bacitracin methylene disalicylate and C) In-water essential oil- chicks supplied the essential oil via the water route at the recommended dosage of 250 mL/1000 L of drinking water; D) *In ovo* saline treatment- eggs injected with 0.2 mL of physiological saline (0.9%

NaCl); E) *In ovo* essential oil treatment- eggs injected with 0.2mL of a saline + essential oil blend mixture at a dilution ratio of 2:1, F) *In ovo* + in-water essential oil treatment- chicks offered the essential oil blend via the *in ovo* and in water route, successively. Gene Ontology terms included both biological process (BP), cellular component (CC), and molecular function (M .
